# Changes in the community structure of the symbiotic microbes of wild amphibians from the eastern edge of the Tibetan Plateau

**DOI:** 10.1002/mbo3.1004

**Published:** 2020-02-11

**Authors:** Liang Liang Xu, Hua Chen, Mengjie Zhang, Wei Zhu, Qing Chang, Guoqing Lu, Youhua Chen, Jianping Jiang, Lifeng Zhu

**Affiliations:** ^1^ Chengdu Institute of Biology Chinese Academy of Sciences Chengdu China; ^2^ College of Life Sciences Nanjing Normal University Nanjing China; ^3^ Shanghai Biozeron Bioinformatics Center Shanghai China; ^4^ Department of Biology University of Nebraska at Omaha Omaha NE USA

**Keywords:** altitude, amphibians, *Bufo gargarizans*, environmental adaptation, gut microbes, skin microbes

## Abstract

Environment has a potential effect on the animal symbiotic microbiome. Here, to study the potential relationship of the symbiotic microbiomes of wild amphibians with altitude, we collected the gut and skin samples from frogs (nine species) and the environmental samples (water and soil samples) from the Leshan Mountains (altitude: 360–410 m) and Gongga Mountains (altitude: 3340–3989 m) on the eastern edge of the Tibetan Plateau. *Bufo gargarizans* (Bg) samples were collected from both the Leshan and Gongga mountain regions (Bg was the only species sampled on both mountains). The DNA extracted from each sample was performed high‐throughput sequencing (MiSeq) of bacterial 16S rRNA gene amplicons. High relative abundance of Caulobacteraceae and Sphingomonadaceae was found in skin samples from both Bg and the other high‐altitude amphibians (nine species combined). High relative abundance of Coxiellaceae and Mycoplasmataceae was found in gut samples from both Bg and the other high‐altitude amphibians. Furthermore, the alpha and beta diversities of skin and gut samples from Bg and the other amphibian species (nine species combined) were similar. In terms of the symbiotic microbial community, the low‐altitude samples were less diverse and more similar to each other than the high‐altitude samples were. We speculated that extreme high‐altitude environments and host phylogeny may affect the amphibian microbiome. Despite the distinct microbial community differences between the skin and gut microbiomes, some functions were similar in the Bg and combined high‐altitude samples. The Bg and high‐altitude skin samples had higher oxidative stress tolerance and biofilm formation than the low‐altitude skin samples. However, the opposite results were observed for the Bg and high‐altitude gut samples. Further study is required to determine whether these characteristics favor high‐altitude amphibian adaptation to extreme environments.

## Graphical abstract

Altitude has a potential effect on the animal symbiotic microbiome. Here, we provided an example of this effect in the wild amphibians. High relative abundance of Caulobacteraceae and Sphingomonadaceae was found in skin samples from both *Bufo gargarizans* and the other high‐altitude amphibians (nine species combined). High relative abundance of Coxiellaceae and Mycoplasmataceae was found in gut samples from both *B. gargarizans* and the other high‐altitude amphibians.

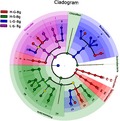

## 1 INTRODUCTION

Symbiotic microbes play important roles in host disease immunity (Hooper, Dan, & Macpherson, 2012) and environmental adaptation (Lavrinienko, Tukalenko, Mappes, & Watts, 2018). The Tibetan Plateau has a unique environment characterized by low temperature, humidity, and air pressure. Many species (mammals, birds, reptiles, and amphibians) inhabit high‐altitude environments (Jungfer, 2011; Zhang, Li, Tang, Liu, & Zhao, 2018; Zhao et al., 2018; Zhou et al., 2016). Research on extreme environment microorganisms has focused on symbiotic organisms harbored by animals inhabiting high altitudes and their ability to adapt to extreme environments. Zhao *et al.* observed that a higher abundance of *Ruminococcaceae* and *Christensenellaceae* may help Chinese rhesus macaques (*Macaca mulatta*) adapt to cellulose‐containing foods and maintain a low body mass index (BMI) to survive in the high‐altitude zone of the plateau (Zhao et al., 2018). The results of previous studies showed that although plateau pika have a lower dietary diversity (types of food) than Daurian pika, the amount of gut microbes (*Prevotella* and *Ruminococcus*) was higher in plateau pika, which could improve their ability to digest plants and increase the observed levels of biodegradation (Li et al., 2018). Taken together, these studies show that an increased abundance of specific gut microorganisms improves the overall digestibility of nutrients in mammals living at high altitude (Li et al., 2018; Zhang et al., 2016; Zhao et al., 2018). The effect of altitude on the gut microbiome has also been investigated in other vertebrates, such as Tibetan chickens (Zhou et al., 2016) and lizards (Zhang et al., 2018).

However, compared to gut microbes, few studies have examined the altitudinal community structure of skin microbiomes on animals, particularly at high altitudes. Some studies of skin microbes showed that the dominance of specific bacterial groups varies with altitude. For example, five skin bacterial genera (including *Arthrobacter*, *Paenibacillus*, and *Carnobacterium*) were shown to be significantly enriched on both humans and pigs living at high altitudes compared to those living at low altitudes (Zeng et al., 2017). These bacterial groups may help humans and pigs adapt more easily to the high‐altitude environment. For example, *Paenibacillus*, which occurs on the skin of high‐altitude humans and pigs, can protect the skin against hypoxia and UV radiation (Zeng et al., 2017). Similarly, the mean relative abundance of Comamonadaceae (0.015% vs. 0.008%), with anti‐*Batrachochytrium dendrobatidis* (Bd) activity, is more abundant in the skin microbiomes of *Silverstoneia flotator* at high altitudes than at low altitudes (Medina et al., 2017). Moreover, compared to gut microbes, skin microbes are believed to be more susceptible to environmental fluctuations (Longo, Savage, Ian, & Zamudio, 2015; Longo & Zamudio, 2017).

Amphibians are well known for their extreme sensitivity to environmental changes compared to other vertebrate groups (Hopkins, 2007). Symbiotic bacteria (i.e., gut and skin microbes) play essential roles in regulating amphibians’ capacity for environmental adaptation (Chang, Huang, Lin, Huang, & Liao, 2016). Amphibian skin is a uniquely selective environment that harbors dominant bacterial groups (Walke et al., 2014). Due to direct contact with the environment, amphibian skin microbes are influenced by various environmental factors (e.g., temperature and moisture) (Longo & Zamudio, 2017; Varela, Lesbarrères, Ibáñez, & Green, 2018). The frog gut microbiome can vary across different habitats, which may help the host utilize food resources more effectively and adapt to environmental changes (Chang et al., 2016; Huang, Chang, Huang, Gao, & Liao, 2017).

The potential relationship between symbiotic microbes (i.e., gut and skin microbes) and altitude is one of the fundamental questions in microbial ecology. However, few studies have systematically compared the community structure of skin and gut microbiomes across altitudinal gradients. Compared with the skin microbe composition of other animals living on plateaus, the composition of amphibian skin microbes may be especially important in helping them adapt to the high‐altitude environment (Zeng et al., 2017). Therefore, a better understanding of amphibian skin and gut microbes can help us better understand and study amphibians living in high‐altitude environments.

To investigate the impact of altitude on the community structure of symbiotic microbiomes of the skin and gut, we examined ten common amphibian species. The water and soil in which these animals lived, which included both high‐ and low‐altitude habitats, were sampled. On the basis of the microbial data derived from high‐throughput sequencing of the bacterial 16S rRNA gene in the gut and skin samples, we asked the following three questions: (a) What are the differences in the skin and gut microbiomes of amphibians living at high versus low altitudes? (b) Which functions are associated with the skin and gut microbiomes of frogs living at different altitudes? (c) What are the potential relationships between the frog symbiotic microbiome and the environmental microbial community (e.g., aquatic microbial community)?

## 2 MATERIALS AND METHODS

### 2.1 Sample collection

Eighty‐eight gut samples, 77 skin samples, and 39 environmental samples (14 water samples and 24 soil samples) were collected from the Leshan and Gongga Mountains in Sichuan Province, China, between May and July 2018 (Table 1). The Leshan Mountains have an average altitude and temperature of 381 m and 20°C, respectively. In contrast, the Gongga Shan Mountains have an average altitude and temperature of 3,557 m and 10°C, respectively. All sampling instruments and materials were sterilized before sampling each individual animal and site. To avoid harming the amphibians, a net capture method was used to collect samples.

**Table 1:** A summary of the characteristics of samples obtained from amphibians living at different altitudes

Location	Scientific name	Species (*n*)	Altitude (*m*)	Time	GPS information	Soil (*n*)	Water (*n*)
Leshan (Low‐altitude)	*Bufo gargarizans*	L‐Bg (8, 15, 1)	380	May	E.103.6405; N.29.4987	21	11
	*Fejervarya limnocharis*	L‐Fl (18, 20, 18)	370	May	E.103.1036; N.29.4569		
	*Pelophylax nigromaculatus*	L‐Pn (12, 14, 11)	385	May	E.103.5998; N.29.4569		
	*Rana omeimontis*	L‐Ro (3, 2, 2)	360	May	E.103.3353; N.29.2713		
	*Microhyla fissipes*	L‐Mf (16, 16, 16)	410	May	E.103.5998; N.29.4569		
Gongga Shan (High‐altitude)	*Bufo gargarizans*	H‐Bg (4, 4, 4)	3,400	July	E.101.3841; N.30.0574	3	3
	*Scutiger glandulatus*	H‐Sg (4, 4, 4)	3,340	July	E.101.5483; N.29.8008		
	*Nanorana parkeri*	H‐Np (4, 4, 4)	3,654	July	E.101.5939; N.29.7868		
	*Amolops kangtingensis*	H‐Ak (4, 4, 4)	3,400	July	E.101.4103; N.29.6733		
	*Batrachuperus tibetanus*	H‐Bt (4, 4, 4)	3,989	July	E.101.5939; N.29.7868		

Note

*n*, the number of samples. The number in the bracket represented the number of skin samples, gut samples, and common samples.

For skin microbial sampling, each animal was rinsed three times with sterile water to remove potential transient bacteria before collecting skin microbes (Lauer et al., 2007). For unified sampling standards, sterile swabs that had no germicidal effects on the microbes were used to rub the back, side, and abdomen of each animal three times. The swabs were then transferred to 2‐ml aseptic centrifuge tubes.

For environmental sampling, each water sample was collected in two 5‐L sterile PET bottles and stored immediately at −20°C (Liu et al., 2018). The collected water samples were filtered using a vacuum pump with a pressure of 0.5 MPa and a membrane aperture and diameter of 0.2 μm and 10 cm, respectively. The filter paper used within the vacuum pump was placed inside a sterile 2‐ml centrifuge tube (Zwart, Crump, Agterveld, Hagen, & Han, 2002). Soil samples (2.5 cm in diameter and 13 cm deep) were collected in triplicate using an aseptic shovel (Chang, Haudenshield, Bowen, & Hartman, 2017). The collected samples were immediately transferred to a sterile self‐sealing bag for preservation. Environmental samples were collected to study the proportion of the aquatic and soil microbial community associated with the symbiotic microorganisms of amphibians.

For gut microorganism sampling, amphibians were euthanized and dissected. The gut contents were collected and immediately transferred to a 2‐ml aseptic centrifuge tube. All samples (skin, water, soil, and gut samples) were immediately frozen at −20°C in a portable refrigerator. After returning to the laboratory, the samples collected in the field were immediately stored at −80°C. All experiments were approved by the Institution of Animal Care and the Ethics Committee of the Chengdu Institute of Biology, Chinese Academy of Sciences.

### 2.2 DNA extraction and sequencing

The samples were thawed for DNA extraction using a QIAamp DNA Stool Mini kit (Qiagen). The V4 region of the 16S rRNA gene was PCR‐amplified using 515F (5′‐GTGCCAGCMGCCGCGG‐TAA‐3′) and 806R (5′‐GACTACHVGGGTWTCTAAT3′) primers (Caporaso et al., 2012). PCR was performed in a 20‐μl volume using 10 ng of the DNA template, 2.5 mM dNTPs, 5 μM of each primer, 5×FastPfu buffer, and FastPfu polymerase. The PCR thermocycling conditions were as follows: initial denaturation at 95°C for 5 min followed by 35 cycles of amplification at 95°C for 30 s, 55°C for 30 s, and 72°C for 45 s, with a final extension step at 72°C for 10 min. The PCR amplification products were sent to Shanghai Lingen Biotechnology Co., Ltd., for high‐throughput sequencing on the Illumina MiSeq platform.

### 2.3 Diversity analysis

The raw sequence data were processed using QIIME1.9 (Caporaso et al., 2010). The function *Trimmomatic* was used for quality control, the function *flash* was used for splicing, and the function *search* was used to detect chimerism to remove low‐quality sequences (Edgar, 2010). All sequences with >97% identity were treated as an operational taxonomic unit (OTU), and each OTU was classified by annotation against the SILVA132 database (Christian et al., 2012).

Gut and skin microbial compositions from animals sampled at different altitudes were compared using the linear discriminant analysis (LDA) effect size (LEfSe) method (Segata et al., 2011). The alpha diversity was calculated using the observed OTU number. We used PERMANOVA (number of permutations: 999) based on dissimilarity matrices (i.e., Bray–Curtis distance, unweighted UniFrac distance, and weighted UniFrac distance) to analyze differences in the community of the skin and gut microbes of *Bufo gargarizans* and other amphibians at different altitudes. The results were visualized using nonmetric multidimensional scaling (NMDS) (Anderson, 2010). PERMANOVA was also performed using the function adonis in the *vegan* package (Dixon, 2003) on the unweighted UniFrac distance to compute an R^2^ value (effect size) to determine the percentage of variation explained by host phylogeny (species) or altitude in QIIME1.9 (Caporaso et al., 2010).

### 2.4 Source‐tracking analysis

To study the proportion of the aquatic and soil microbial communities that overlapped with the gut and skin microbes of amphibians at high and low altitudes, Source‐Tracker 0.9.5 was used to assess the correlation between the high‐ and low‐altitude samples of skin, gut contents, water, and soil based on a Bayesian algorithm (Dan et al., 2011). This software is typically used to explore pollution sources and sinks and is therefore appropriate for the analysis of the proportion of the environmental microbial community in the symbiotic microorganism communities of amphibians in this study. The aquatic and soil microbial communities were treated as sources of environmental microbes, while the gut and skin microbiomes were treated as sinks in this analysis.

### 2.5 BugBase analysis

A tool in an R package was used to classify samples into different microbial groups. Group classifications included Gram‐positive, Gram‐negative, biofilm‐forming, potentially pathogenic, mobile element‐containing, oxygen‐utilizing, and oxidative stress‐tolerant bacteria (Tonya, Jake, & Jeremy, 2017). We primarily evaluated the oxidative stress‐tolerant and biofilm‐forming functions of bacteria among the groups (H‐G, gut microbes at high altitude; L‐G, gut microbes at low altitude; H‐S, skin microbes at high altitude; and L‐S, skin microbes at high altitude) (Tonya et al., 2017).

### 2.6 Statistical analysis

In this study, we used the Kruskal–Wallis H test (abbreviation: H) and Mann–Whitney U test (abbreviation: U) to test for significant differences in the abundance of microbial groups (i.e., phylum, family, and genus levels) between the skin and gut microbes of *B. gargarizans* (Bg) and other amphibians at high and low altitudes. We used the Kruskal–Wallis H test and Mann–Whitney U test to test the significance of differences in the microbial alpha and beta diversity between the skin and gut microbes of Bg and other amphibians at high and low altitudes. In the BugBase analysis, we used the Mann–Whitney U test to test the significance of the difference in the proportion of each bacterial function between the high‐ and low‐altitude samples in either Bg or other amphibians (high‐altitude amphibians: *Scutiger glandulatus*, *Nanorana parkeri*, *Amolops kangtingensis*, *Batrachuperus tibetanus* and *B. gargarizans*; low‐altitude amphibians: *Fejervarya limnocharis*, *Pelophylax nigromaculatus*, *Rana omeimontis*, *Microhyla fissipes*, and *B. gargarizans*).

## 3 RESULTS

### 3.1 Composition and comparison of the symbiotic microbiome

**Figure 1:**
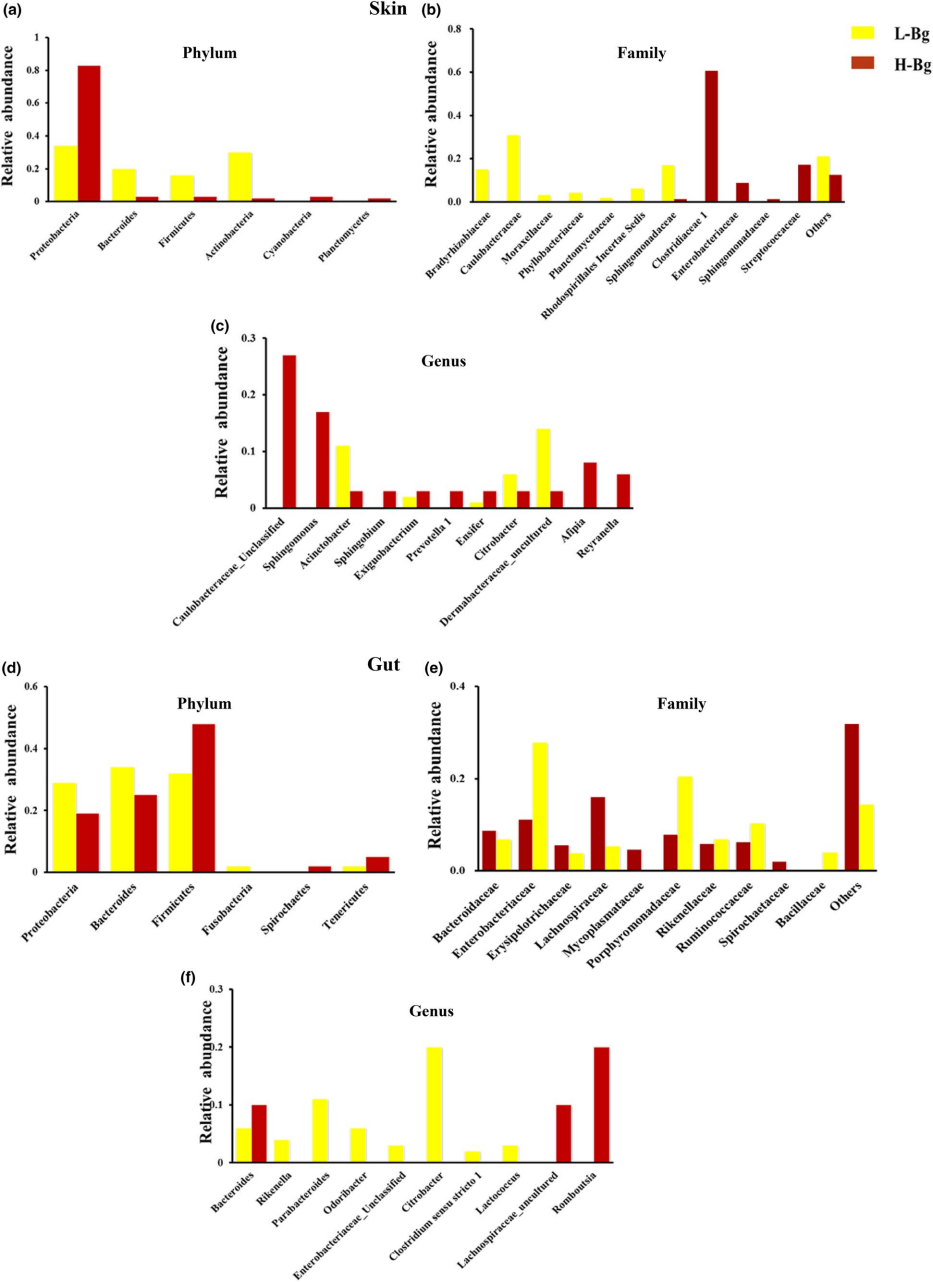
Symbiotic microbiome of *Bufo gargarizans* living at different altitudes. Histogram of the skin microbiota of *B. gargarizans* living at high and low altitudes at the phylum, family, and genus levels (a, b, c). Histogram of the gut flora of *B. gargarizans* living at high and low altitudes at the phylum, family, and genus levels (d, e, f)

**Figure A1:**
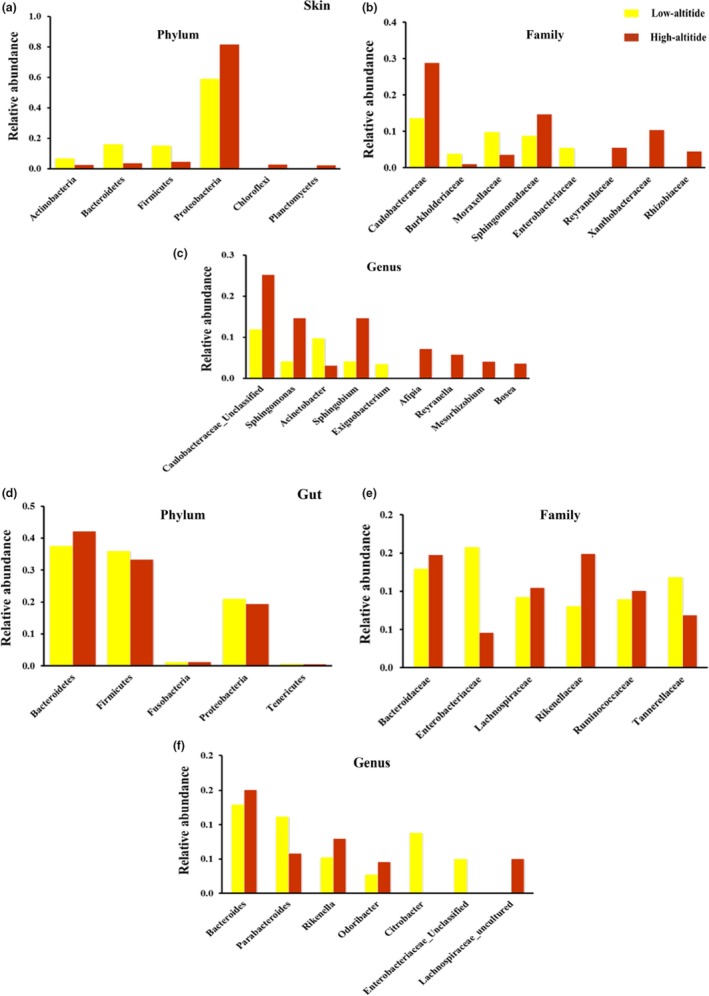
Symbiotic microbiome of amphibians living at different altitudes. Histogram of skin obtained from different phyla, families, and genera of amphibians living at high or low altitudes (a, b, c); histogram of intestinal flora at the phylum, family, and genus level of amphibians living at high or low altitudes (d, e, f)

**Figure A2:**
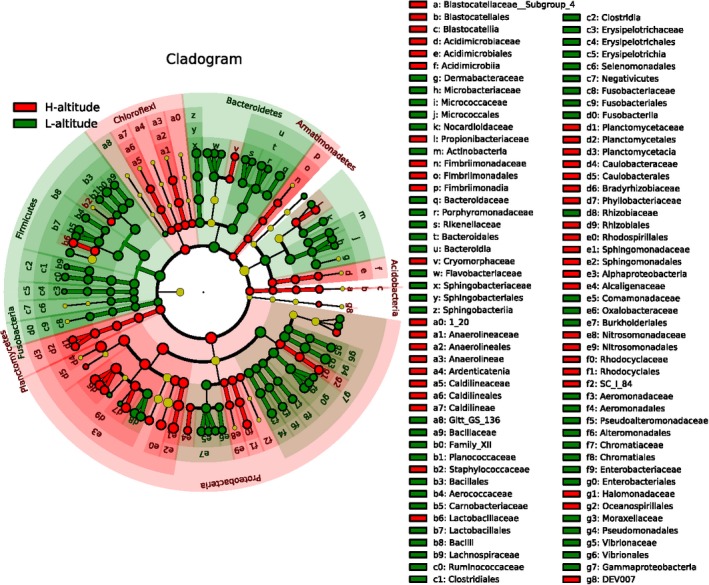
Linear discriminant analysis effect size (LEfSe) analysis of skin microbiome in amphibians living at high and low altitudes. Histogram of the LDA scores computed for differentially abundant features between low‐altitude and high‐altitude gut microbes. H‐, high altitude; L‐, low altitude. From the outside to the inside, the red‐ and green‐colored nodes represent bacteria of the phylum, class, order, family, genus, and species, which display significant differences. The yellow‐colored nodes represent the bacteria displaying no significant difference

After conducting quality control and filtering processes, we obtained 4,381,379 high‐quality sequences from the skin, gut, water, and soil samples collected at high and low altitudes. The average number of sequences per sample was 22,476. The high‐ and low‐altitude amphibian symbiotic microbiome (skin and gut) were composed of 459 OTUs across eight phyla (Figure A1 in Appendix 2; Tables A1, A2, A3, A4, A5, A6 in Appendix 1). There were differences in the composition of microbes on the skin or in the gut at high versus low altitudes. In the skin samples, the abundances of Proteobacteria, Bacteroidetes, and Firmicutes were significantly different between high and low altitude for Bg (distributed at high and low altitudes) (both U, *p* < .05) and other amphibians (including Bg) (both, H, *p* < .001) (Table 2; Figure A2 in Appendix 2). In the gut samples from high and low altitudes, there was no significant altitudinal difference in the abundances of Proteobacteria, Bacteroidetes, and Firmicutes for Bg (both U, *p* > .05); whereas significant differences were observed in the abundance of Proteobacteria, Bacteroidetes were observed with altitude for the other amphibians (both, H, *p* < .05) (Table 2; Figure A2 in Appendix 2). In the water or soil samples, we also observed some common significant differences in the composition of microbiome between the low‐ and high‐altitude samples. For example, the abundance of Proteobacteria was significantly different between the L‐Water (the aquatic microbial community at low altitude) and H‐Water (the aquatic microbial community at high altitude) samples (U, *p* < .05) and between the L‐Soil (the soil microbial community at low altitudes) and H‐Soil (the soil microbial community at high altitudes) samples (U, *p* < .05) (Figure A4 in Appendix 2; Tables A7, A8, A9, A10, A11, A12 in Appendix 1). Interestingly, we observed that in the skin microbes, the abundance of the families and genera belonging to Proteobacteria, except for *Exiguobacterium*, showed significant differences between the low‐ and high‐altitude samples (Figure 1b,c in Appendix 2). In the gut samples, the abundances of Proteobacteria, Bacteroides, and Firmicutes were significantly higher at high altitudes than at low altitudes (Figure 1d in Appendix 2).

**Table 2:** Significant differences in the skin and intestinal microbiome of *Bufo* *gargarizans* and amphibians (including all species in this study) between high and low elevations (phylum level)

Altitude and sample		Phylum		
		Proteobacteria	Bacteroidetes	Firmicutes
Skin	L‐Bg	***	***	*
	H‐Bg			
	Low amphibians	***	***	***
	High amphibians			
Gut	L‐Bg	NS	NS	NS
	H‐Bg			
	Low amphibians	**	***	NS
	High amphibians			

Note

*p* > .05 marked as “NS”; *p* < .05 marked as “*”; *p* < .01 marked as “**”; *p* < .001 marked as “***”; “L‐Bg”: *B. gargarizans* at low altitude; “H‐Bg”: *B. gargarizans* at high altitude; “Low amphibians”: other amphibians at low altitude; “High amphibians”: other amphibians at high altitude. We used the Mann–Whitney U test and Kruskal–Wallis H test to test for significant differences in the abundance of phyla between the skin and gut microbiomes of Bg and other amphibians.

### 3.2 Comparison of the skin microbes on Bg and other amphibians at high altitudes

**Figure 2:**
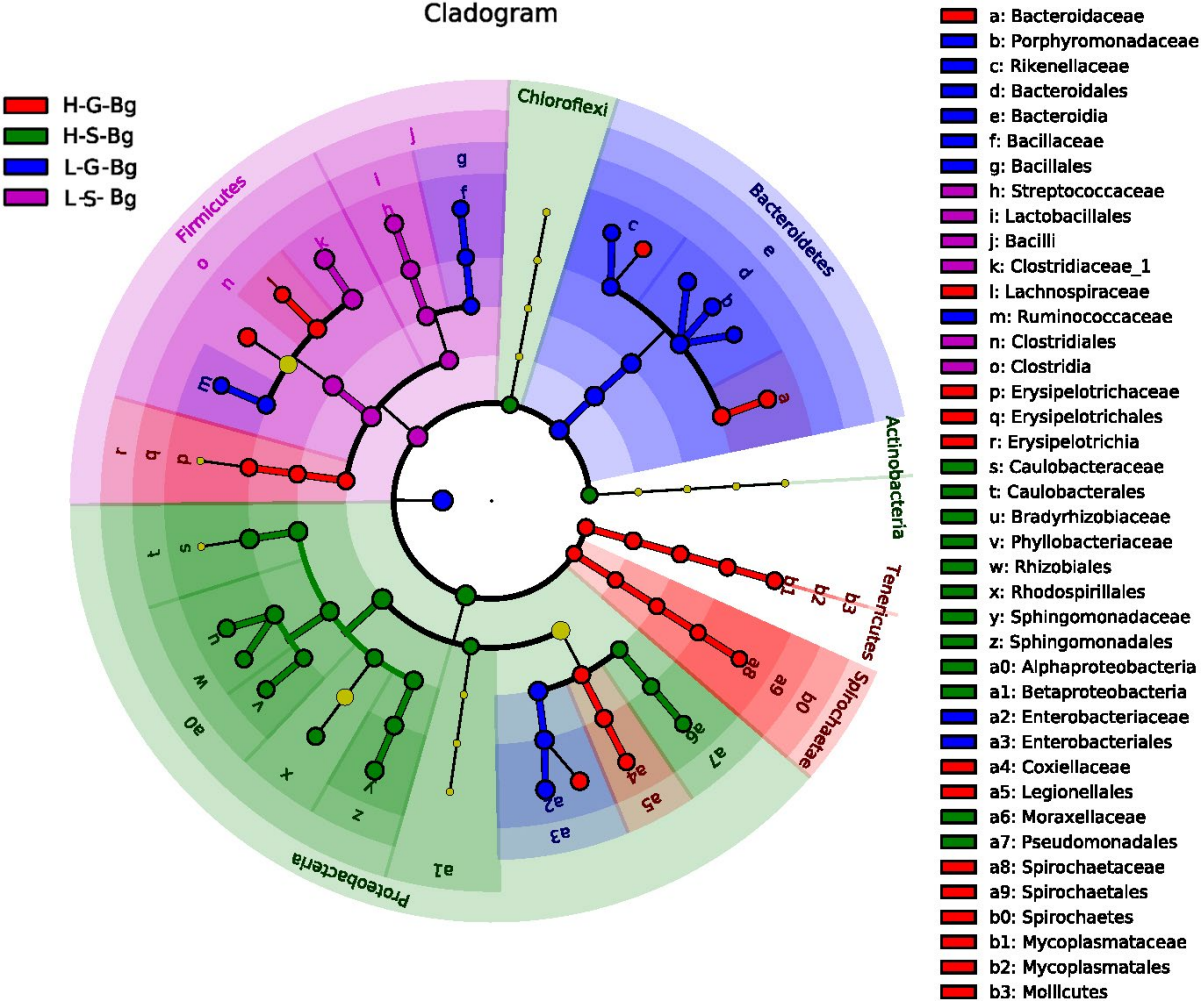
Linear discriminant analysis effect size (LEfSe) of the skin and gut microbiome in *Bufo gargarizans* living at high and low altitudes. Cladogram of the LDA scores computed for differentially abundant features between low‐ and high‐altitude skin microbiomes. H‐, high altitude; L‐, low altitude; S‐, skin microbes; G‐, gut microbes; Bg (*B. gargarizans*). From the outside to the inside, the red‐ and green‐colored nodes represent the bacteria that displayed significant differences at the phylum, class, order, family, genus, and species levels. The yellow‐colored nodes represent bacteria displaying no significant difference

In skin samples, the five LEfSe families (Caulobacteraceae, Bradyrhizobiaceae, Phyllobacteriaceae, Sphingomonadaceae, and Moraxellaceae) were significantly enriched in the H‐S‐Bg samples (the skin samples from Bg at high altitudes), with observed relative abundances of 31%, 15%, 4%, 17%, and 3%, respectively (Figure 2). In the H‐S samples (total level: all skin samples from all species at high altitudes combined), fifteen families were significantly enriched, including Planctomycetaceae (2%), Caulobacteraceae (29%), and Sphingomonadaceae (15%) (Figure A2 in Appendix 2). Interestingly, Caulobacteraceae and Sphingomonadaceae were detected in both the H‐S‐Bg and H‐S samples. With respect to the H‐S‐Bg samples, the relative abundance of Caulobacteraceae in the H‐Bg samples was higher than that in the L‐Bg samples (U, *p* < .01), while the relative abundance of Caulobacteraceae in the H‐S samples was higher than that in the L‐S samples (U, *p* < .001).

### 3.3 Comparison of the skin microbes on Bg and other amphibians at low altitudes

The L‐S‐Bg samples (species level: skin samples from Bg at low altitudes) were enriched in two bacterial families (Clostridiaceae 1 and Streptococcaceae), with relative abundances of 61% and 17%, respectively (Figure 2). The L‐S samples (total level: all skin samples from all species at low altitudes combined) were enriched in 25 families (Figure A2 in Appendix 2), including Micrococcaceae (1%), Bacteroidaceae (2%), Porphyromonadaceae (2%), Clostridiaceae 1 (29%), and Rikenellaceae (2%). In addition, Clostridiaceae 1 was enriched in both the L‐S‐Bg and L‐S samples. In the L‐S‐Bg samples, the relative abundance of Clostridiaceae 1 was greater than that in the H‐S‐Bg samples (U, *p* < .01) but similar to that in the L‐S samples (U, *p* < .001).

### 3.4 Comparison of the gut microbes of Bg and amphibians at high altitudes

**Figure A3:**
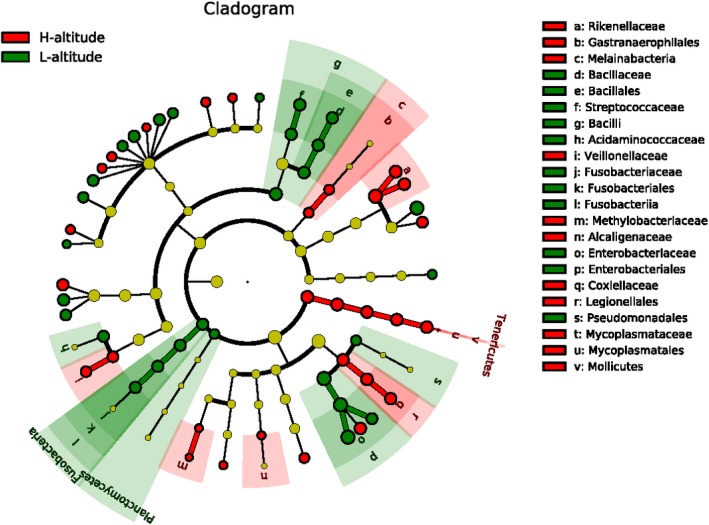
Linear discriminant analysis effect size (LEfSe) analysis of intestinal microbiome in amphibians living at high and low altitudes. Histogram of the LDA scores computed for differentially abundant features between low‐altitude and high‐altitude gut microbes. H‐, high altitude; L‐, low altitude. From the outside to the inside, the red‐ and green‐colored nodes represent bacteria of the phylum, class, order, family, genus, and species, which display significant differences. The yellow‐colored nodes represent the bacteria displaying no significant difference

**Figure A4:**
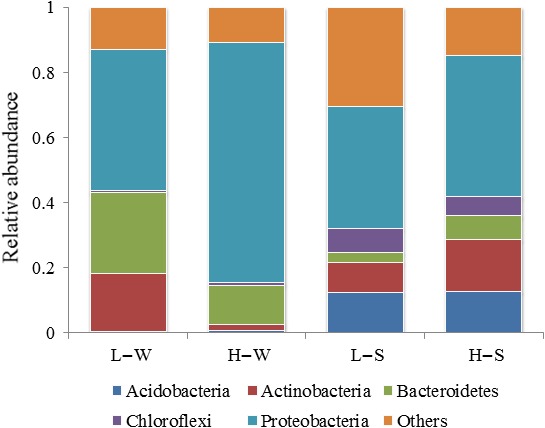
Composition of microbiome at phylum level in environmental samples at high and low altitudes. L‐W: low‐altitude water samples; L‐S: low‐altitude soil samples; H‐W: high‐altitude water samples; H‐S: high‐altitude soil samples

In the gut samples, the H‐G‐Bg samples (the gut samples from Bg at high altitudes) were enriched in six bacterial families (Bacteroidaceae, Lachnospiraceae, Erysipelotrichaceae, Coxiellaceae, Spirochaetaceae, and Mycoplasmataceae), with relative abundances of 9%, 16%, 6%, 8%, 2%, and 5%, respectively (Figure 2). The H‐G samples (total level: all gut samples from all species at high altitudes combined) were also enriched in six bacterial families (Rikenellaceae, Veillonellaceae, Methylobacteriaceae, Alcaligenaceae, Coxiellaceae, and Mycoplasmataceae), with relative abundances of 15, 1, 3, 1, 2, and 2%, respectively (Figure A3 in Appendix 2). Coxiellaceae and Mycoplasmataceae were enriched in the H‐G‐Bg and H‐G samples. For example, in the H‐G‐Bg samples, the relative abundance of Mycoplasmataceae was higher than that in the L‐G‐Bg samples (U, *p* < .05), while in the H‐G samples, the relative abundance of Mycoplasmataceae was higher than that in the L‐G samples (U, *p* > .05).

### 3.5 Comparison of the gut microbes of Bg and other amphibians at low altitudes

The L‐G‐Bg samples (the gut samples from Bg at low altitudes) were enriched in five bacterial families (Enterobacteriaceae, Porphyromonadaceae, Rikenellaceae, Ruminococcaceae, and Bacillaceae), with relative abundances of 28%, 20%, 7%, 10%, and 4%, respectively (Figure 2). The L‐G samples (total level: all gut samples from all species at low altitudes combined) were also enriched in five bacterial families (Bacillaceae, Streptococcaceae, Acidaminococcaceae, Fusobacteriaceae, and Enterobacteriaceae), with relative abundances of 2, 5, 1, 2, and 17%, respectively (Figure A3 in Appendix 2). In addition, Enterobacteriaceae and Bacillaceae were enriched in both the L‐G‐Bg and L‐G samples. For example, in the L‐G‐Bg samples, the relative abundance of Enterobacteriaceae was higher than that in the H‐G‐Bg samples (U, *p* < .05), while in the L‐G samples, the relative abundance of Enterobacteriaceae was higher than that in the H‐G‐Bg samples (U, *p* < .001).

In addition, we statistically analyzed the compositional differences in the skin and gut microbiota of Bg and other amphibians at the same altitude. The proportion of Firmicutes and Proteobacteria was significantly different (both, U, *p* < .05) between the skin and gut microbiomes of the H‐Bg samples (*B. gargarizans* at high altitude) (Table A14 in Appendix 1). Only three genera (*Acinetobacter*, *Sphingomonas*, and an unclassified *Caulobacteraceae* genus) showed a significant difference (both U, *p* < .05) between the L‐S‐Bg and L‐G‐Bg samples (Table A16 in Appendix 1). However, in general (in the amphibians), the two bacterial phyla, Firmicutes and Proteobacteria, also showed significant differences (both, H, *p* < .001) in the skin and gut microorganisms present within amphibians from the same altitudes (Table 3; Table A13 in Appendix 1). In addition, the proportions of six common genera were also significantly different (both, H, *p* < .001) between the skin and gut microbiomes within amphibians from the same altitude (Table A15 in Appendix 1) (*Bacteroides*, *Acinetobacter*, *Sphingomonas*, *Afipia*, *Mesorhizobium*, and *Rikenella*).

**Table 3:** Significantly different microbiome in the skin and gut contents of *Bufo gargarizans* and amphibians (including all species in this study) between high and low elevations (genus level)

Altitude and sample		Bacterium					
		*Acinetobacter*	*Sphingomonas*	*Caulobacteraceae_* *Unclassified*	*Bacteroides*	*Parabacteroides*	*Rikenella*
Skin	L‐Bg	*	***	***	NS	NS	NS
	H‐Bg						
	Low amphibians	**	***	***	NS	NS	NS
	High amphibians						
Gut	L‐Bg	NS	NS	NS	NS	NS	NS
	H‐Bg						
	Low amphibians	NS	NS	NS	**	**	**
	High amphibians						

Note

*p* > .05 marked as “NS”; *p* < .05 marked as “*”; *p* < .01 marked as “**”; *p* < .001 marked as “***”; “L‐Bg”: *B. gargarizans* at low altitude; “H‐Bg”: *B. gargarizans* at high altitude; “Low amphibians”: other amphibians at low altitude; “High amphibians”: other amphibians at high altitude. We used the Mann–Whitney U test and Kruskal–Wallis H test to test for significant differences in the abundances of genera between the skin and gut microbes of Bg and amphibians.

### 3.6 Differences in the alpha and beta diversities of the amphibian microbiome

**Figure 3:**
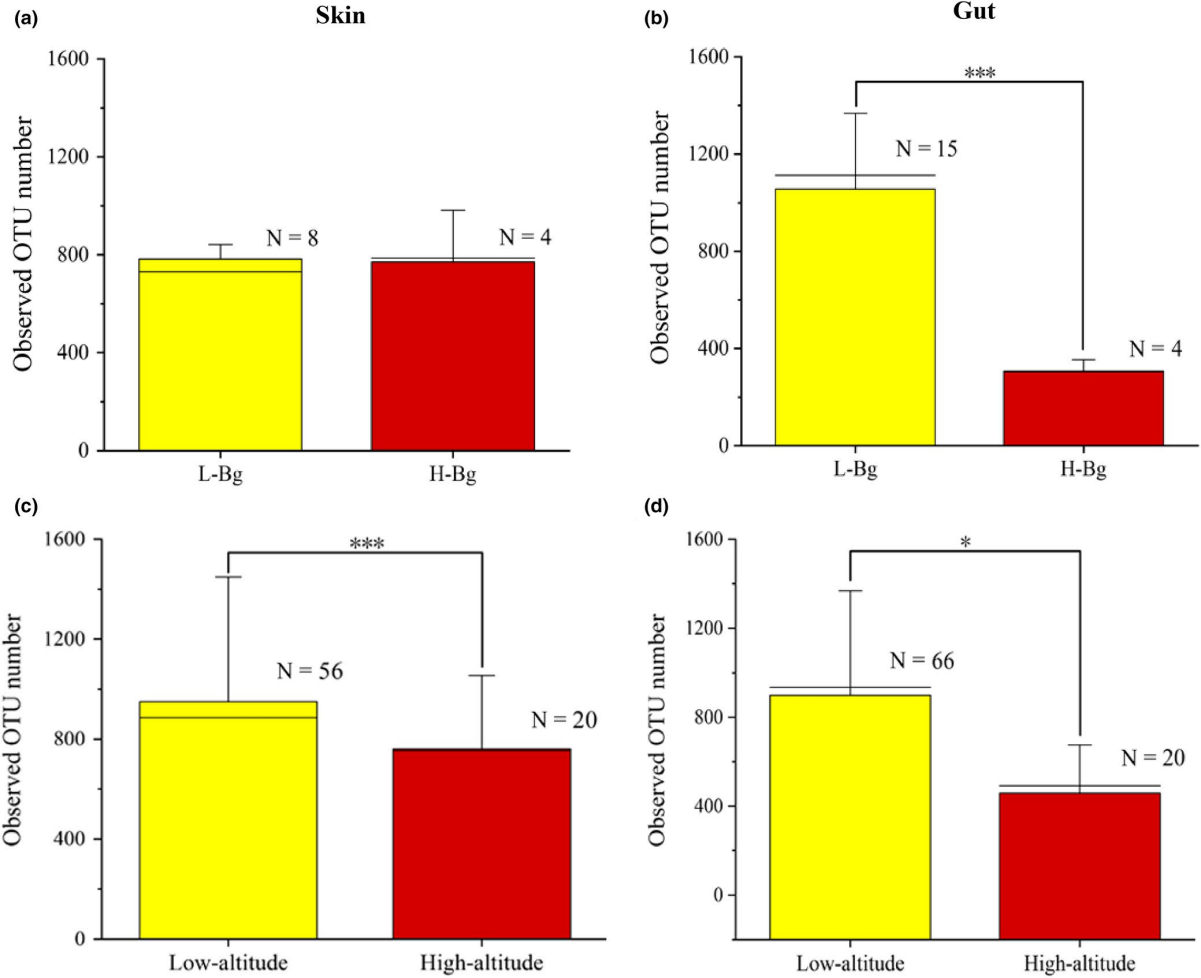
The alpha diversity of symbiotic microbiomes from *Bufo gargarizans* and amphibians living at different altitudes. Comparisons of the observed OTU number (average) for the skin microbes (a) and gut microbes (b) between low‐ and high‐altitude *B. gargarizans*. Comparisons of the observed OTU number (average) for the skin microbes (b) and gut microbes (d) between low‐ and high‐altitude amphibians (including all species in this study). The Mann–Whitney U test was used to test the differences between groups (*p < .05; ***p < .001). The error bars represent the standard deviation, and the long horizontal black line represents the link function, indicating the two samples involved in the comparison (*n*: represents sample size)

**Figure 4:**
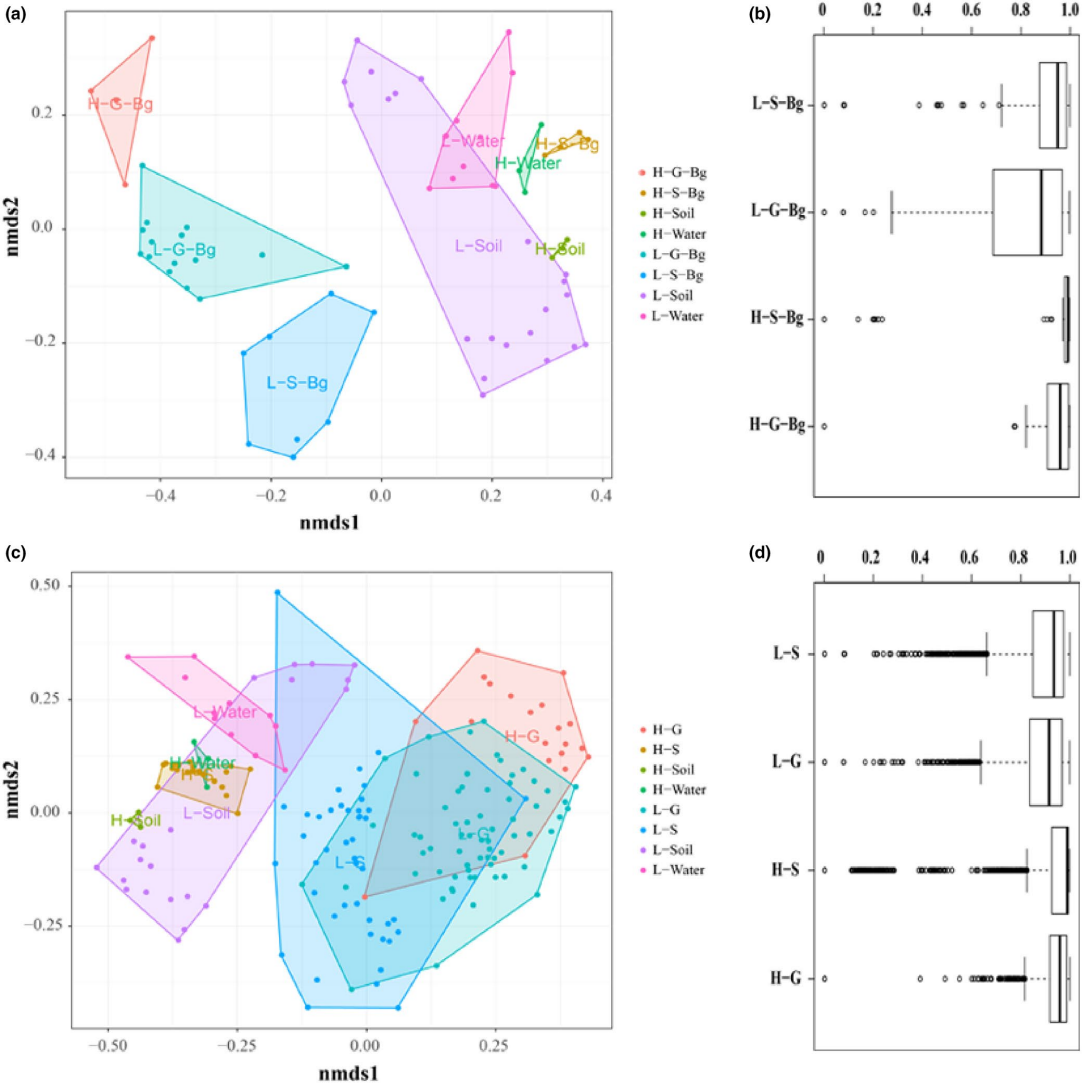
Effects of altitude on the symbiotic microbial community. The dissimilarities (Bray–Curtis distance) among the symbiotic microbiomes of *Bufo gargarizans* (a) and amphibians (c) living at different altitudes were quantified using nonmetric multidimensional scaling (NMDS). The summarized dissimilarity within the same type of symbiotic microbiome for *B. gargarizans* (b) and amphibians (d). H‐G, high‐altitude gut samples; H‐S, high‐altitude skin samples; H‐S‐Bg, high‐altitude *B. gargarizans* skin samples; H‐G‐Bg, high‐altitude *B. gargarizans* gut samples. H‐Soil, high‐altitude soil samples; H‐Water, high‐altitude water samples; L‐G, low‐altitude gut samples; L‐S, low‐altitude skin samples; L‐S‐Bg, low‐altitude *B. gargarizans* skin samples; L‐G‐Bg, low‐altitude *B. gargarizans* gut samples; L‐Soil, low‐altitude soil samples; L‐Water, low‐altitude water samples

**Figure A5:**
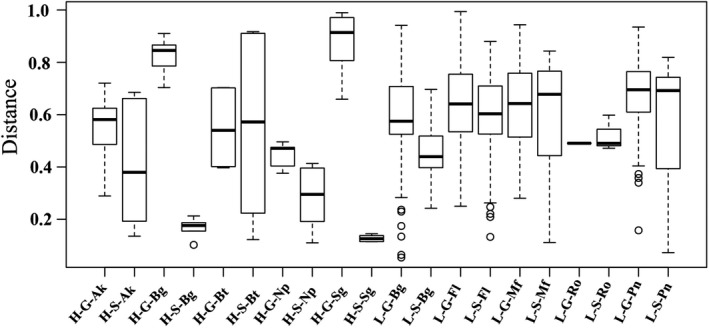
Bray–Curtis distances of skin and intestinal microbiome based on different species at high and low elevations: H‐G‐Ak, H‐S‐Ak, H‐G‐Bg, H‐S‐Bg, H‐G‐Bt, H‐S‐Bt, H‐G‐Np, H‐S‐Np, H‐G‐Sg, H‐S‐Sg, L‐G‐Bg, L‐S‐Bg, L‐G‐Fl, L‐S‐Fl, L‐G‐Mf, L‐S‐Mf, L‐G‐Ro, L‐S‐Ro, L‐G‐Pn, L‐S‐Pn, respectively (H‐, represents high altitude; L‐, represents low altitude; G‐, represents gut microbes; S‐, represents skin microbes; Ak, represents *Amolops kangtingensi*; Bg, represents *Bufo gargarizans*; Bt, represents *Batrachuperus tibetanus*; Np, represents *Nanorana parkeri*; Sg, represents *Scutiger glandulatus*; Fl, represents *Fejervarya limnocharis*; Mf, represents *Microhyla fissipes*; Ro, represents *Rana omeimontis*; Pn, represents *Pelophylax nigromaculatus*)

**Figure A6:**
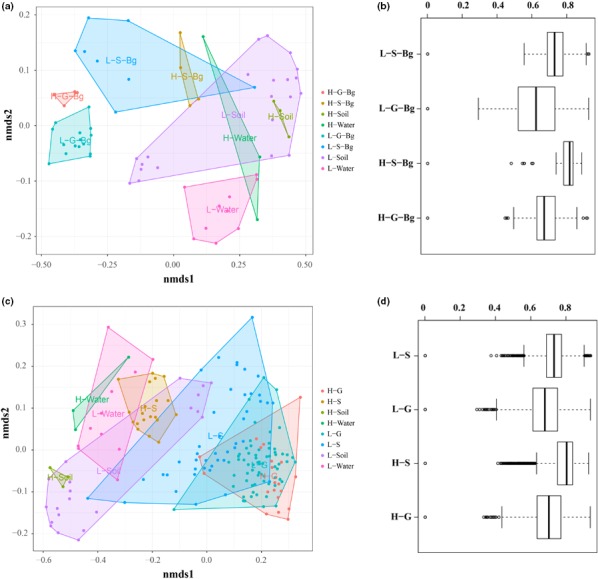
The dissimilarities (unweighted_unifrac distance) among the symbiotic microbiome of *Bufo gargarizans* (a) and all species (c) living at different altitudes were quantified using nonmetric multidimensional scaling (NMDS). The summarized dissimilarity within the same type of symbiotic microbiome for *Bufo gargarizans* (b) and all species (d). H‐G, high‐altitude gut samples; H‐S, high‐altitude skin samples; H‐S‐Bg, high‐altitude *Bufo gargarizans* skin samples; H‐G‐Bg, high‐altitude *Bufo gargarizans* gut samples. H‐Soil, high‐altitude soil samples; H‐Water, high‐altitude water samples; L‐G, low‐altitude gut samples; L‐S, low‐altitude skin samples; L‐S‐Bg, low‐altitude *Bufo gargarizans* skin samples; L‐G‐Bg, low‐altitude *Bufo gargarizans* gut samples; L‐Soil, low‐altitude soil samples; L‐Water, low‐altitude water samples

**Figure A7:**
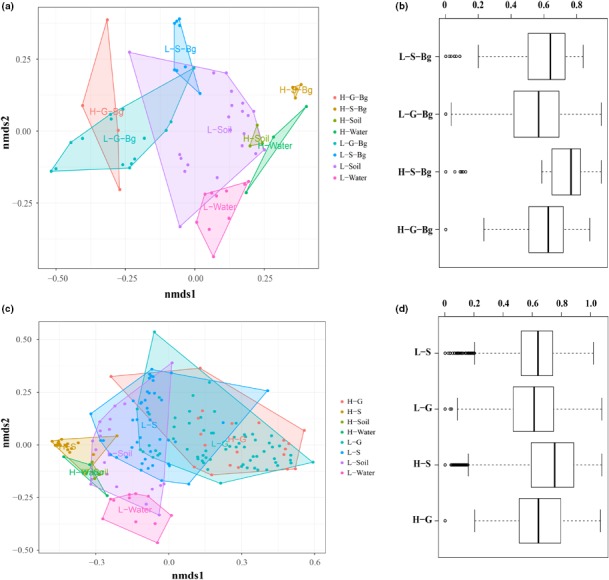
The dissimilarities (weighted_unifrac distance) among the symbiotic microbiome of *Bufo gargarizans* (a) and all species (c) living at different altitudes were quantified using nonmetric multidimensional scaling (NMDS). The summarized dissimilarity within the same type of symbiotic microbiome for *Bufo gargarizans* (b) and all species (d). H‐G, high‐altitude gut samples; H‐S, high‐altitude skin samples; H‐S‐Bg, high‐altitude *Bufo gargarizans* skin samples; H‐G‐Bg, high‐altitude *Bufo gargarizans* gut samples. H‐Soil, high‐altitude soil samples; H‐Water, high‐altitude water samples; L‐G, low‐altitude gut samples; L‐S, low‐altitude skin samples; L‐S‐Bg, low‐altitude *Bufo gargarizans* skin samples; L‐G‐Bg, low‐altitude *Bufo gargarizans* gut samples; L‐Soil, low‐altitude soil samples; L‐Water, low‐altitude water samples

**Figure A8:**
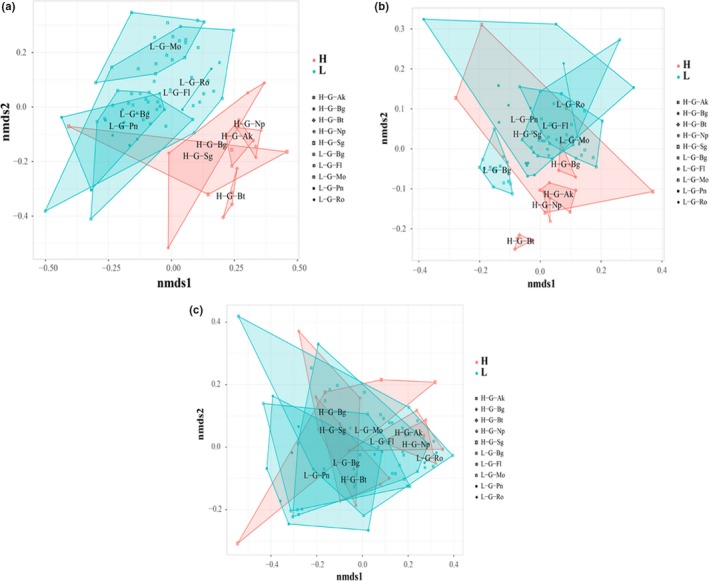
The dissimilarities on bray_curtis distance (a), unweighted _unifrac distance (b), and weighted_unifrac distance (c) among the gut microbiome of *Bufo gargarizans* and other species living at different altitudes were quantified using nonmetric multidimensional scaling (NMDS). H‐G‐Ak, H‐S‐Ak, H‐G‐Bg, H‐S‐Bg, H‐G‐Bt, H‐S‐Bt, H‐G‐Np, H‐S‐Np, H‐G‐Sg, H‐S‐Sg, L‐G‐Bg, L‐S‐Bg, L‐G‐Fl, L‐S‐Fl, L‐G‐Mf, L‐S‐Mf, L‐G‐Ro, L‐S‐Ro, L‐G‐Pn, L‐S‐Pn, respectively (H‐, represents high altitude; L‐, represents low altitude; G‐, represents gut microbes; S‐, represents skin microbes; Ak, represents *Amolops kangtingensi*; Bg, represents *Bufo gargarizans*; Bt, represents *Batrachuperus tibetanus*; Np, represents *Nanorana parkeri*; Sg, represents *Scutiger glandulatus*; Fl, represents *Fejervarya limnocharis*; Mf, represents *Microhyla fissipes*; Ro, represents *Rana omeimontis*; Pn, represents *Pelophylax nigromaculatus*)

**Figure A9:**
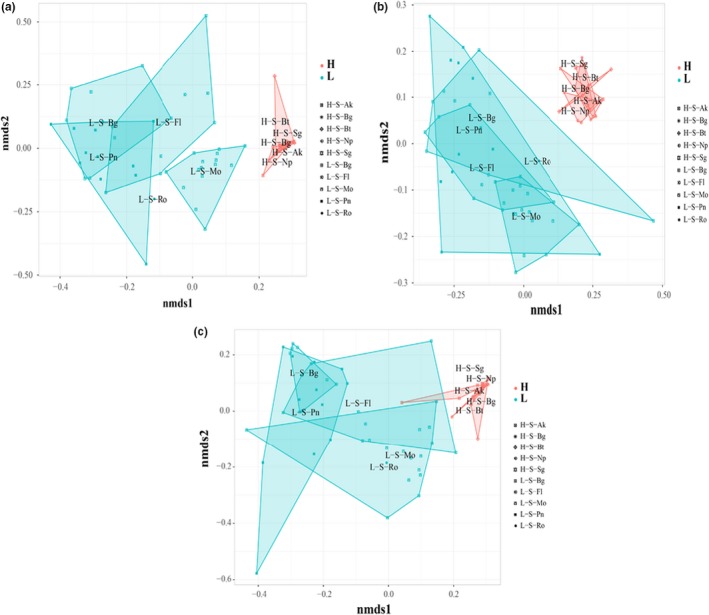
The dissimilarities on bray_curtis distance (a), unweighted _unifrac distance (b), and weighted_unifrac distance (c) among the skin microbiome of *Bufo gargarizans* and other species living at different altitudes were quantified using nonmetric multidimensional scaling (NMDS). H‐G‐Ak, H‐S‐Ak, H‐G‐Bg, H‐S‐Bg, H‐G‐Bt, H‐S‐Bt, H‐G‐Np, H‐S‐Np, H‐G‐Sg, H‐S‐Sg, L‐G‐Bg, L‐S‐Bg, L‐G‐Fl, L‐S‐Fl, L‐G‐Mf, L‐S‐Mf, L‐G‐Ro, L‐S‐Ro, L‐G‐Pn, L‐S‐Pn, respectively (H‐, represents high altitude; L‐, represents low altitude; G‐, represents gut microbes; S‐, represents skin microbes; Ak, represents *Amolops kangtingensi*; Bg, represents *Bufo gargarizans*; Bt, represents *Batrachuperus tibetanus*; Np, represents *Nanorana parkeri*; Sg, represents *Scutiger glandulatus*; Fl, represents *Fejervarya limnocharis*; Mf, represents *Microhyla fissipes*; Ro, represents *Rana omeimontis*; Pn, represents *Pelophylax nigromaculatus*)

The L‐G‐Bg samples had significantly higher OTU numbers than the H‐G‐Bg samples (U, *p* < .01) (Figure 3a,b). Low‐altitude samples had a significantly higher observed OTU number than the high‐altitude samples for both skin (H, *p* < .01) and gut (H, *p* < .05) samples (Figure 3c,d). NMDS using three types of distances showed that the compositions of microbial communities in the Bg and other amphibian samples were different and could be easily distinguished between high and low altitudes, especially the skin samples (Figure 4; Tables A6, A7 in Appendix 1; Figures A6, A7, A8, A9 in Appendix 2). Both altitude (*p* < .01, PERMANOVA) and amphibian species (*p* = .001) (Bray–Curtis distance and unweighted UniFrac distance) had significant effects on the skin and gut microbial community structure of Bg and other amphibians (Tables 4 and 5; Figure A5 in Appendix 2). Thus, in addition to the host phylogeny (species), the differences in the microbiomes in this study were also strongly affected by altitude. Moreover, for the skin microbiomes, the similarity in the bacterial community structure of the high‐altitude samples was greater than that of the low‐altitude samples (similar among only Bg samples or only other amphibian samples). However, this trend was not observed for the gut microbiomes (Figure 4b,d).

**Table 4:** PERMANOVA results for the gut and skin microbiome obtained from *Bufo gargarizans* living at high and low altitudes

Sample	PERMANOVA					
	Type	Distance	*df*	*F*	*R* ^2^	*p* Value
Gut	Altitude	Bray_Curtis	1	3.5331	.17207	.001
		Unweighted_UniFrac	1	5.4099	.24141	.001
		Weighted_UniFrac	1	1.9432	.10258	.054
Skin	Altitude	Bray_Curtis	1	8.6314	.46327	.004
		Unweighted_UniFrac	1	3.1978	.2423	.005
		Weighted_UniFrac	1	26.984	.72961	.002

**Table 5:** PERMANOVA results for the gut and skin microbiome obtained from amphibians living at high and low altitudes

Sample	PERMANOVA					
	Type	Distance	*df*	*F*	*R* ^2^	*p* Value
Gut	Altitude	Bray_Curtis	1	5.3484	.06365	.001
		Unweighted_UniFrac	1	4.2326	.05025	.001
		Weighted_UniFrac	1	1.551	.01902	.14
	Species	Bray_Curtis	7	4.0124	.27512	.001
		Unweighted_UniFrac	7	3.9507	.27205	.001
		Weighted_UniFrac	7	3.0736	.22525	.001
Skin	Altitude	Bray_Curtis	1	16.761	.24026	.001
		Unweighted_UniFrac	1	10.293	.16263	.001
		Weighted_UniFrac	1	24.426	.31548	.001
	Species	Bray_Curtis	7	3.8042	.36167	.001
		Unweighted_UniFrac	7	2.5291	.27361	.001
		Weighted_UniFrac	7	5.524	.45137	.001

### 3.7 Functional predictions of the symbiotic microbiome

**Figure 5:**
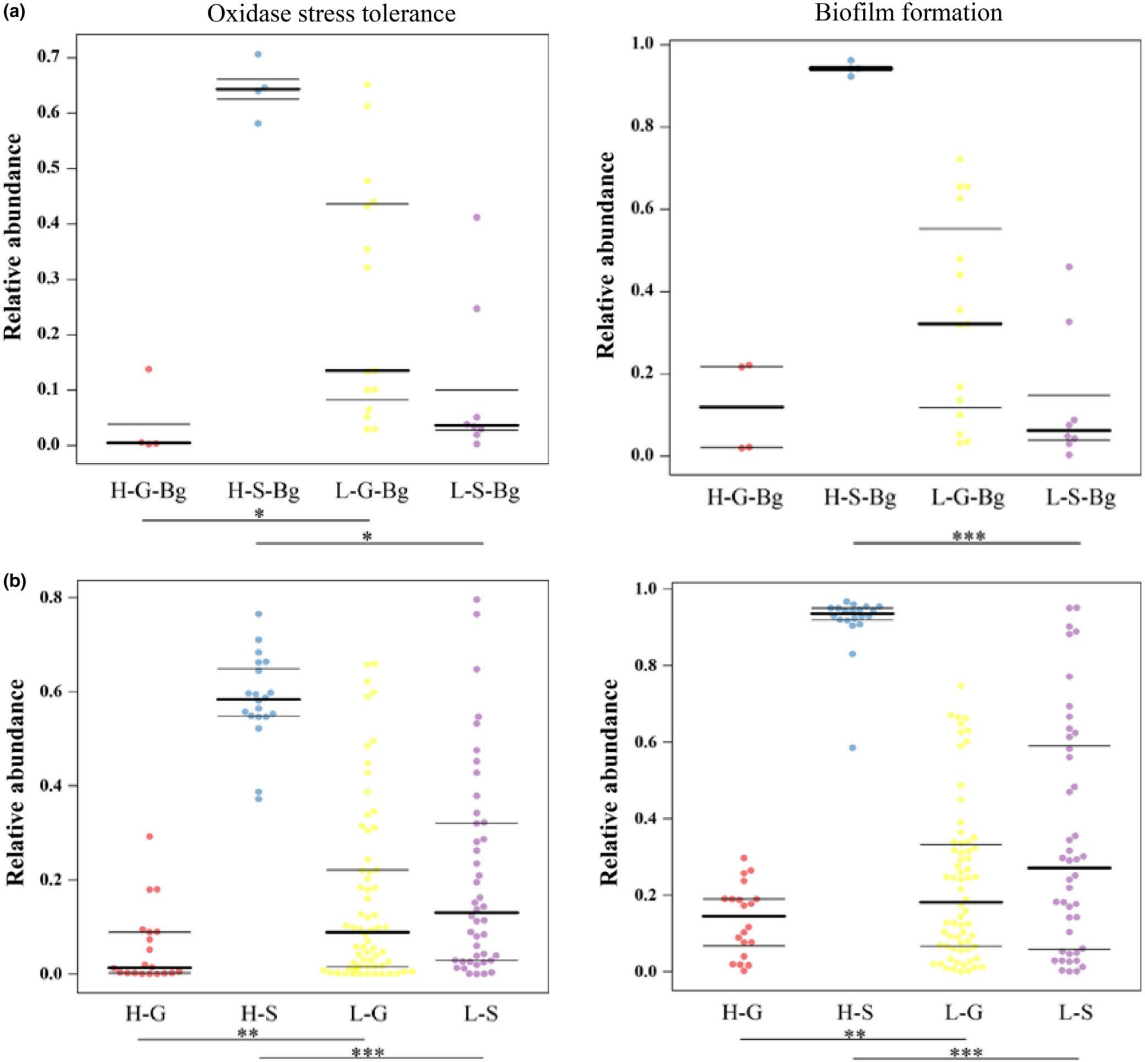
Putative oxygen‐related functions enriched in symbiotic microbiomes. H‐, high altitude; L‐, low altitude. Black lines represent quartiles. The statistical test is based on nonparametric differentiation tests (Mann–Whitney U test). *p < .05, **p < .01; ***p < .001. Each spot represents one sample. (a) For *Bufo gargarizans*; (b) for amphibians including all species in this study

The putative functions (for oxidative stress tolerance and biofilm formation) of the skin microbiomes for the high‐ and low‐altitude Bg samples were significantly different (both, *p* < .05) (Figure 5a). For amphibians (including all species in this study), the high‐altitude skin samples exhibited a greater level of biofilm formation than the low‐altitude skin samples (Figure 5b). However, the oxidative stress tolerance was higher in only the H‐S‐Bg samples and was lower in the H‐S samples (Figure 5b).

### 3.8 Correlation between symbiotic and environmental microbiomes

**Figure 6:**
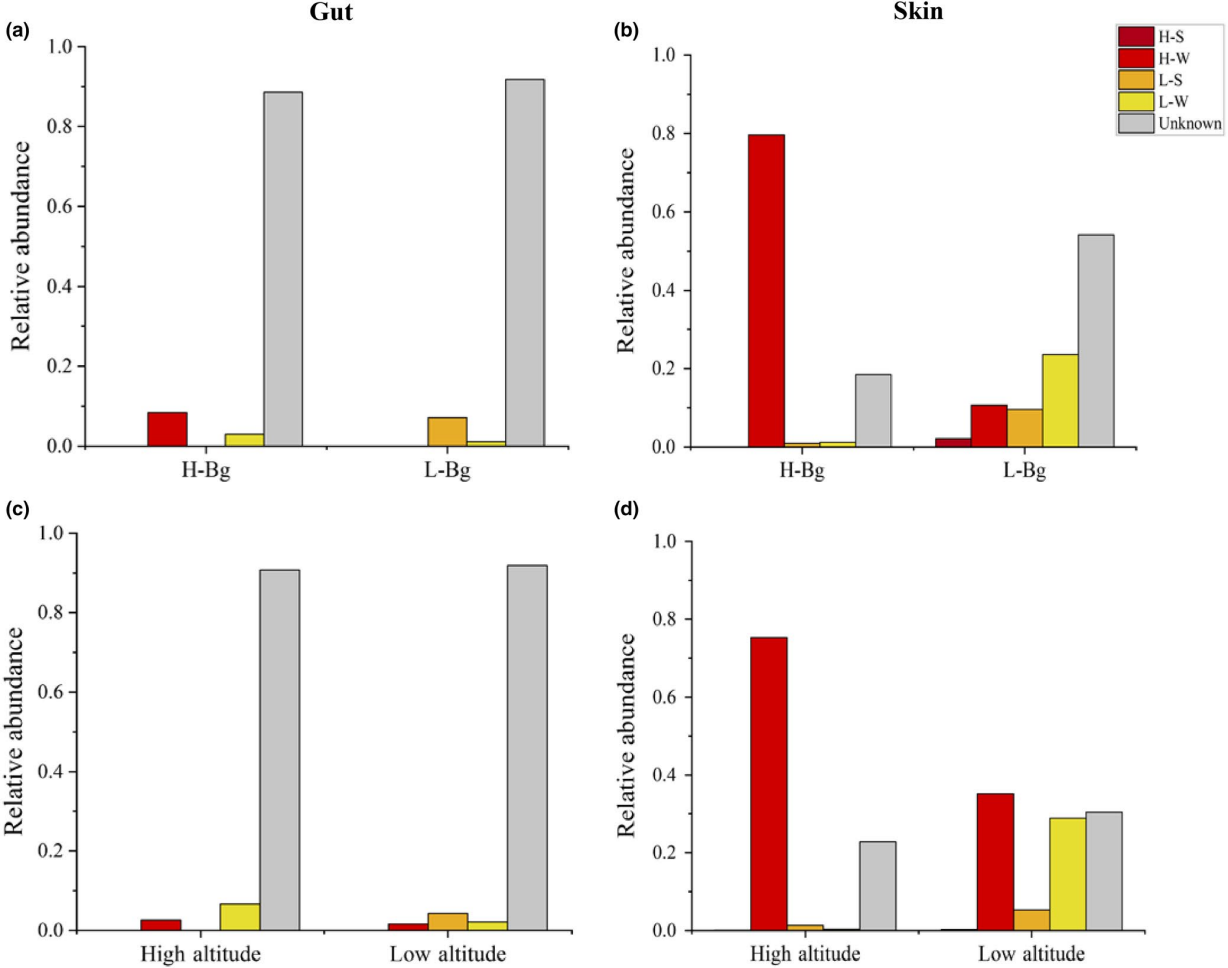
The correlation between symbiotic microbiome in amphibians and the surrounding environmental microbiome. Source‐tracking analysis of skin microbes b, d), gut microorganisms (a, c), and the environmental microbiome in *Bufo gargarizans* and amphibians (including all species in this study) living at high and low elevations. The value on the *x*‐axis is the predicted proportion of the source microbiomes in the sink samples. H‐S, high‐altitude soil; H‐W, high‐altitude water; L‐S, low‐altitude soil; L‐W, low‐altitude water. Unknown (unknown source): the portion of a sink sample that is unlikely to be assigned to any of the known sources in this study may come from other unknown sources (Knights *et al.,* 2011); H‐Bg: *B. gargarizans* at high altitude; L‐Bg: *B. gargarizans* at low altitude. The value on the *y*‐axis represents the relative abundance of the samples. Each graph represents a microbial sample, and the height of the color histogram represents the proportion of each source in the samples

The results of the source‐tracker analysis revealed that a high number of skin microbes present in all amphibians (or only in Bg) at high and low elevations were from the aquatic microbial community (Figure 6b,d). Bacteria from the H‐S (high‐altitude soil microbial community) and L‐S (low‐altitude soil microbial community) samples were rarely from the aquatic microbial community, while the gut microbes were almost never from the aquatic and soil microbial communities (Figure 6a,c). The NMDS results also supported this finding (Figure 4; Figures A6 and A7 in Appendix 2).

## 4 DISCUSSION

In this study, we showed that both host phylogeny and altitude have a significant effect on the amphibian microbial community (especially in the composition and structure of the skin microbiome). The results obtained for Bg, the only species sampled at all sites, mirrored the patterns observed across all species and showed that altitude and host species significantly affected host microbiomes. After comparing the beta diversity results for the gut microbiome, the only instance of this pattern not being observed was for the weighted UniFrac distance, which may reflect the similarity in the gut microbiome community at the weighted (quantitative) level between the low‐ and high‐altitude samples. In addition, changes in the microbiomes between the high‐ and low‐altitude amphibians predictably led to differences in bacterial oxidative stress tolerance and biofilm formation.

### 4.1 Putative relationship between the composition of microbial groups and the altitude environment

Environmental factors have profound effects on the skin and gut microbial composition of amphibians (Das et al., 2018; Zeng et al., 2017). In our study, the host species likely influenced the amphibian skin and gut microbial compositions, with a more significant impact observed on the skin microbiome (Table 2). We observed many significantly enriched microbes in the skin samples, which may reflect selection pressure from the high‐altitude environment. For example, we detected significantly enriched microbes in the H‐S‐Bg samples, including members of the bacterial families Sphingomonadaceae, Caulobacteraceae, Bradyrhizobiaceae, Sphingomonadaceae, Phyllobacteriaceae, and Moraxellaceae (Figures 1 and 2), among which Caulobacteraceae and Sphingomonadaceae are commonly present in water (Stovepoindexter & Cohen, 1964; White, Sutton, & Ringelberg, 1996). Considering the relatively high proportion of water‐originating microbes in the H‐Bg skin samples, we speculated that in high‐altitude environments, Bg may be more likely to live in an aquatic environment than Bg at low‐altitude environments.

In addition, we observed commonalities between H‐G‐Bg and H‐G in the taxa that were significantly enriched in the gut microbial communities. Mycoplasmataceae was also previously detected in the guts of animals (i.e., salmon) (Holben, Williams, Saarinen, Särkilahti, & Apajalahti, 2002; Llewellyn et al., 2015; Macpherson, 1963). The abundance of Mycoplasmataceae may reflect changes in the host environment. For example, in Atlantic salmon (*Salmo salar*), the gut microbiome of marine adults with a higher abundance of Mycoplasmataceae was less rich and diverse than that of freshwater juveniles (Llewellyn et al., 2015). In our study, the relative abundance of Mycoplasmataceae was higher in the H‐G‐Bg and H‐G samples (5% in H‐G‐Bg vs. 0.1% in L‐G‐Bg; 2% in H‐G vs. 0.1% in L‐G). This may be related to the effect of a high‐altitude environment on gut microorganisms in amphibians (or in Bg alone). In addition, the relative abundance of Coxiellaceae was higher in the H‐G‐Bg and H‐G samples (8% in H‐G‐Bg vs. 0.1% in L‐G‐Bg; 2% in H‐G vs. 0.1% in L‐G).

The skin microorganisms on Bg and other amphibians were similar to those of the aquatic microbial community that they inhabited. Thus, amphibians may obtain these bacteria from the aquatic environment through skin contact (Walke et al., 2014), and high‐altitude environments may affect the symbiotic bacteria of amphibians. Although different amphibian species were sampled at high and low altitudes, Bg was sampled at all altitudes and still showed skin and gut microbiome compositional differences, suggesting that some differences can be attributable to altitude and not just to differences in host species composition.

### 4.2 High‐altitude environments may contribute to the decrease in alpha and beta diversities in symbiotic microbiomes

In our study, the observed OTU numbers were lower in both the skin and gut microbiomes of amphibians at high altitude (or in Bg alone), similar to the OTU values observed in humans, pigs, and Chinese rhesus macaques at high and low altitudes (Zeng et al., 2017; Zhao et al., 2018). The environment can affect symbiotic microbial diversity (skin and gut microbes) in amphibians (Chang et al., 2016; Wolz, Yarwood, Grant, Fleischer, & Lips, 2017). From low to high elevations, host habitats change, often toward increased ultraviolet radiation and a lower oxygen content and temperature. These factors may affect the skin and gut microbes of host organisms (Das et al., 2018; Zeng et al., 2017). Thus, higher altitudes represent a complex natural selective pressure on the symbiotic microbiomes present in animals and the environment. Moreover, we observed that the similarities in the skin microbial communities within high‐altitude species were significantly greater than those within the low‐altitude species (Figure 4). Therefore, high‐altitude environments may be an important selective pressure on the symbiotic microbiomes of amphibians. Although different amphibian species were present at high and low altitudes, which may have impacted the observed alpha and beta diversities, both high‐ and low‐altitude Bg samples showed similar results. These results suggest that the differences between the observed alpha and beta diversities were also affected by altitude, not just by species.

### 4.3 Predicted functions of symbiotic bacteria may reflect adaptations to high‐altitude environments

The symbiotic microbiomes (skin and gut microbiomes) detected in Bg and the other amphibians at high altitudes likely have convergent functions. A significantly higher proportion of bacteria were observed to be associated with biofilm formation in the H‐S‐Bg and H‐S samples than in the L‐S‐Bg and L‐S samples, respectively. Biofilm formation may be a protective mechanism through which bacteria can withstand extreme environments (low temperature, low or high pH, and strong ultraviolet light exposure) and reproduce (César, Fany, Lucía, & Robert, 2013; Stoodley, Sauer, Davies, & costerton JW, 2002). Therefore, it is possible that high‐altitude, skin‐residing microorganisms adapt to the extreme environment of the plateau by forming a biofilm (Schommer & Gallo, 2013). In addition, although the skin and gut microbiomes represent distinct ecological niches and amphibians harbor different microbial communities, both of these microbiomes were observed to have different functions in this study. Compared with the H‐S samples, the H‐S‐Bg samples had a higher level of oxidative stress tolerance, whereas the H‐G‐Bg and H‐G samples exhibited little difference in this function. These results show that the H‐S‐Bg microbiome had high oxygen tolerance and could adapt well to the low‐oxygen environment at high altitude. However, specific mechanisms need to be further studied in the future. The primary focus of this study was to assess the bacterial skin and gut compositions of high‐altitude amphibians and their importance in the ability of amphibians to adapt to this extreme environment. However, these results could also improve the understanding of theoretical basis for other studies to evaluate adaptation to extreme environments.

## 5 CONCLUSIONS

Both altitude and host species may exert significant selective pressure on the composition of skin and gut microbes detected in Bg and other amphibians. We found that the skin microbiome of Bg living at high altitudes had more bacterial groups from the surrounding aquatic environment than the skin microbes of L‐Bg and the gut microbes of L‐Bg and H‐Bg had. We speculate that this may be related to the preference of Bg for the aquatic environment at high altitudes. The skin and gut microorganisms detected in the high‐altitude samples had some common patterns: low alpha diversity and higher proportion of biofilm‐forming phenotypes. These features may play a role in the environmental adaptation of amphibians. However, specific adaptation mechanisms still need to be studied. Thus, the interaction between animals and their symbiotic microbiomes is interesting and is complicated by differences in their habitats. In the future, to improve our understanding of this interaction in wild species, additional ecological factors that may contribute to this phenomenon should be investigated, such as host taxonomy, behavior, and physiology (e.g., habitat selection, life history, phylogeny, and feeding behavior).

## CONFLICT OF INTEREST

None declared.

## AUTHOR CONTRIBUTIONS

Liangliang Xu: Methodology‐Equal, Resources‐Equal, Writing‐original draft‐Equal, Writing‐review & editing‐Equal. Hua Chen: Formal analysis‐Equal, Methodology‐Equal, Software‐Equal. Mengjie Zhang: Data curation‐Equal, Resources‐Equal. Wei Zhu: Methodology‐Equal, Resources‐Equal. Qing Chang: Methodology‐Equal, Resources‐Equal. Guoqing Lu: Methodology‐Equal, Resources‐Equal. Youhua Chen: Data curation‐Equal, Resources‐Equal. Jiangping Jiang: Conceptualization‐Equal, Funding acquisition‐Equal, Methodology‐Equal, Resources‐Equal. Lifeng Zhu: Conceptualization‐Equal, Data curation‐Equal, Formal analysis‐Equal, Funding acquisition‐Equal, Investigation‐Equal, Methodology‐Equal, Project administration‐Equal, Resources‐Equal, Software‐Equal, Supervision‐Equal, Validation‐Equal, Visualization‐Equal, Writing‐original draft‐Equal, Writing‐review & editing‐Equal.

## ETHICS STATEMENT

The animal use protocol for this study (permit: 2017‐AR‐JJP‐03) was reviewed and approved by the Animal Ethical and Welfare Committee of the Chengdu Institute of Biology, Chinese Academy of Science, Chengdu, 610041, China.

## Data Availability

Sequencing data and relevant files are available in the NCBI repository under the BioPoject number PRJNA549036 (https://www.ncbi.nlm.nih.gov/bioproject/PRJNA549036).

## APPENDIX 1

**Table A1:** The composition of skin microbiome at phylum level at high and low altitude

Altitude	Species	Skin microbiota composition (relative abundance)					
		Proteobacteria	Bacteroides	Firmicutes	Actinobacteria	Cyanobacteria	Planctomycetes
Low altitude	L‐Bg	0.34	0.20	0.16	0.30	0.00	0.00
	L‐Fl	0.65	0.12	0.17	0.03	0.01	0.00
	L‐Pn	0.72	0.10	0.13	0.04	0.00	0.00
	L‐Mf	0.52	0.26	0.16	0.03	0.01	0.00
	L‐Ro	0.75	0.09	0.11	0.04	0.01	0.00
High altitude	H‐Bg	0.83	0.03	0.03	0.02	0.03	0.02
	H‐Np	0.80	0.04	0.04	0.04	0.03	0.02
	H‐Sg	0.87	0.03	0.02	0.02	0.03	0.02
	H‐Ak	0.74	0.04	0.13	0.03	0.02	0.03
	H‐Bt	0.84	0.05	0.02	0.02	0.03	0.02

Abbreviations: L‐/H‐Bg: the *Bufo gargarizans* at low or high altitude; L‐/H‐Fl: the *Fejervarya limnocharis* at low or high altitude; L‐/H‐Pn: the *Fejervarya limnocharis* at low or high altitude; L‐/H‐Mf: the *Microhyla fissipes* at low or high altitude; L‐/H‐Ro; the *Rana omeimontis* at low or high altitude.

**Table A2:** Composition of skin microbiome at family level at high and low altitude

OTU ID	Low altitude (relative abundance)					High altitude (relative abundance)				
	L‐Bg	L‐Fl	L‐Pn	L‐Mf	L‐Ro	H‐Bg	H‐Np	H‐Sg	H‐Bt	H‐Ak
Sphingomonadaceae	0.01	0.04	0.13	0.05	0.07	0.17	0.15	0.17	0.11	0.14
Caulobacteraceae	0.001	0.18	0.14	0.19	0.16	0.31	0.29	0.35	0.24	0.27
Burkholderiaceae	0.01	0.05	0.05	0.02	0.06	0.01	0.01	0.01	0.01	0
Moraxellaceae	0	0.1	0.09	0.11	0.1	0.03	0.04	0.01	0.04	0.05
Clostridiaceae 1	0.61	0.32	0.43	0.12	0.01	0.001	0	0	0	0
Streptococcaceae	0.17	0.04	0.04	0.01	0.01	0	0	0	0	0
Enterobacteriaceae	0.09	0.05	0.06	0.03	0.06	0	0	0	0	0
Planctomycetaceae	0	0	0	0	0	0.02	0.11	0.12	0.09	0.1
Rhodospirillales Incertae Sedis	0	0	0	0	0	0.06	0.04	0.05	0.05	0
Phyllobacteriaceae	0	0	0	0	0	0.04	0.05	0.07	0.06	0.1
Bradyrhizobiaceae	0	0	0	0	0	0.15	0.04	0.04	0.03	0
Others	0.12	0.22	0.06	0.47	0.53	0.23	0.28	0.18	0.37	0.34

**Table A3:** Composition of skin microbiome at genus level at high and low altitude

OTU ID	Low altitude (relative abundance)					High altitude (relative abundance)				
	L‐Bg	L‐Fl	L‐Pn	L‐Mf	L‐Ro	H‐Bg	H‐Np	H‐Sg	H‐Ak	H‐Bt
*Caulobacteraceae_Unclassified*	0.00	0.16	0.07	0.17	0.13	0.27	0.25	0.30	0.23	0.21
*Sphingomonas*	0.00	0.04	0.05	0.05	0.07	0.17	0.14	0.17	0.14	0.11
*Acinetobacter*	0.11	0.10	0.08	0.11	0.13	0.03	0.03	0.01	0.04	0.04
*Sphingobium*	0.00	0.00	0.19	0.00	0.00	0.03	0.03	0.01	0.04	0.04
*Exiguobacterium*	0.02	0.07	0.01	0.03	0.00	0.03	0.03	0.01	0.04	0.04
*Prevotella 1*	0.00	0.00	0.00	0.10	0.00	0.03	0.03	0.01	0.04	0.04
*Ensifer*	0.01	0.04	0.01	0.02	0.08	0.03	0.03	0.01	0.04	0.04
*Citrobacter*	0.06	0.02	0.03	0.01	0.04	0.03	0.03	0.01	0.04	0.04
*Dermabacteraceae_uncultured*	0.14	0.00	0.00	0.00	0.00	0.03	0.03	0.01	0.04	0.04
*Afipia*	0.00	0.00	0.00	0.00	0.00	0.08	0.08	0.08	0.06	0.06
*Reyranella*	0.00	0.00	0.00	0.00	0.00	0.06	0.05	0.07	0.05	0.06
*Halomonas*	0.00	0.00	0.00	0.00	0.00	0.00	0.00	0.00	0.00	0.06
Others	0.66	0.56	0.57	0.52	0.55	0.22	0.24	0.28	0.22	0.23

**Table A4:** Composition of intestinal microbiome at phylum level at high and low altitude

Altitude	Species	Gut microbiota composition (relative abundance)					
		Proteobacteria	Bacteroides	Firmicutes	Fusobacteria	Spirochaetes	Tenericutes
Low altitude	L‐Bg	0.29	0.34	0.32	0.02	0.00	0.02
	L‐Fl	0.12	0.48	0.35	0.00	0.00	0.00
	L‐Pn	0.36	0.21	0.35	0.05	0.00	0.00
	L‐M0	0.14	0.38	0.44	0.00	0.00	0.00
	L‐Ro	0.06	0.70	0.21	0.00	0.00	0.00
High altitude	H‐Bg	0.19	0.25	0.48	0.00	0.02	0.05
	H‐Np	0.06	0.65	0.26	0.00	0.00	0.00
	H‐Sg	0.12	0.29	0.36	0.00	0.09	0.09
	H‐Ak	0.10	0.61	0.25	0.00	0.00	0.03
	H‐Bt	0.23	0.42	0.30	0.00	0.00	0.00

**Table A5:** Composition of intestinal microbiome at family level at high and low altitude

OTU ID	Low altitude (relative abundance)					High altitude (relative abundance)				
	L‐Bg	L‐Fl	L‐Pn	L‐Mf	L‐Ro	H‐Bg	H‐Np	H‐Sg	H‐Ak	H‐Bt
Bacteroidaceae	0.07	0.17	0.08	0.16	0.12	0.09	0.17	0.16	0.25	0.07
Enterobacteriaceae	0.28	0.07	0.28	0.1	0.19	0.11	0	0.09	0.03	0
Erysipelotrichaceae	0.04	0.08	0.03	0.1	0.06	0.06	0.05	0.06	0.05	0.01
Lachnospiraceae	0.05	0.11	0.04	0.15	0.09	0.16	0.03	0.09	0.09	0.14
Mycoplasmataceae	0.001	0.02	0.03	0.01	0.02	0.05	0.01	0.04	0.09	0.05
Rikenellaceae	0.07	0.13	0.05	0.07	0.06	0.06	0.28	0.04	0.19	0.17
Ruminococcaceae	0.1	0.08	0.04	0.15	0.09	0.06	0.11	0.14	0.08	0.11
Porphyromonadaceae	0.2	0.15	0.05	0.11	0.08	0.08	0.16	0.03	0.08	0.06
Bacillaceae	0.04	0	0	0	0	0	0	0	0.04	0.2
Spirochaetaceae	0	0	0	0	0.02	0.02	0.09	0	0	0
Others	0.14	0.18	0.4	0.17	0.27	0.32	0.09	0.34	0.11	0.21

**Table A6:** Composition of intestinal microbiome at genus level at high and low altitude

OTU ID	Low altitude (relative abundance)					High altitude (relative abundance)				
	L‐Bg	L‐Fl	L‐Pn	L‐Mf	L‐Ro	H‐Bg	H‐Np	H‐Sg	H‐Ak	H‐Bt
*Bacteroides*	0.06	0.17	0.08	0.16	0.33	0.1	0.2	0.2	0.2	0.1
*Rikenella*	0.04	0.08	0.03	0.04	0.03	0.0	0.1	0.0	0.1	0.1
*Parabacteroides*	0.11	0.14	0.05	0.11	0.31	0.0	0.2	0.0	0.1	0.0
*Odoribacter*	0.06	0.02	0.03	0.01	0.00	0.0	0.0	0.0	0.1	0.1
*Enterobacteriaceae_Unclassified*	0.03	0.02	0.10	0.05	0.01	0.00	0.00	0.00	0.00	0.00
*Citrobacter*	0.20	0.03	0.15	0.02	0.02	0.00	0.00	0.00	0.00	0.00
*Clostridium *sensu* stricto 1*	0.02	0.02	0.07	0.00	0.01	0.00	0.00	0.00	0.00	0.00
*Lactococcus*	0.03	0.00	0.05	0.00	0.00	0.00	0.00	0.00	0.00	0.00
*Lachnospiraceae_uncultured*	0.00	0.00	0.00	0.00	0.00	0.1	0.0	0.1	0.0	0.1
*Aeromonas*	0.00	0.00	0.00	0.00	0.00	0.0	0.0	0.0	0.0	0.2
*Mucinivorans*	0.00	0.00	0.00	0.00	0.00	0.0	0.1	0.0	0.1	0.0
*Romboutsia*	0.00	0.00	0.00	0.00	0.00	0.2	0.0	0.0	0.0	0.0
Others	0.45	0.51	0.45	0.61	0.30	0.60	0.41	0.69	0.39	0.42

**Table A7:** Composition of water microbiome at phylum level at high and low altitude

Altitude	Samples	Water microbiota composition ‐ (relative abundance)						
		Proteobacteria	Bacteroidetes	Firmicutes	Actinobacteria	Cyanobacteria	Planctomycetes	Verrucomicrobia
Low altitude	L‐W1	0.37	0.07	0.00	0.13	0.42	0.00	0.00
	L‐W2	0.43	0.16	0.01	0.25	0.06	0.02	0.03
	L‐W3	0.40	0.16	0.01	0.27	0.07	0.02	0.02
	L‐W4	0.30	0.37	0.09	0.20	0.01	0.01	0.01
	L‐W5	0.35	0.26	0.01	0.33	0.01	0.01	0.02
	L‐W6	0.34	0.34	0.01	0.30	0.00	0.00	0.00
	L‐W7	0.50	0.26	0.03	0.10	0.02	0.01	0.01
	L‐W8	0.43	0.36	0.02	0.16	0.00	0.00	0.00
	L‐W9	0.44	0.31	0.08	0.12	0.01	0.00	0.00
	L‐W10	0.56	0.24	0.03	0.05	0.02	0.00	0.01
	L‐W11	0.66	0.22	0.03	0.05	0.01	0.00	0.00
High altitude	H‐W1	0.67	0.10	0.04	0.02	0.03	0.03	0.00
	H‐W2	0.67	0.21	0.01	0.02	0.01	0.02	0.00
	H‐W3	0.87	0.05	0.01	0.01	0.01	0.01	0.00

**Table A8:** Composition of water microbiome at family level at high and low altitude

Altitude	Samples	Water microbiota composition (relative abundance)						
		Burkholderiaceae	Caulobacteraceae	Flavobacteriaceae	Sphingomonadaceae	Microbacteriaceae	Methylophilaceae	Beijerinckiaceae
Low altitude	L‐W1	0.26	0.00	0.02	0.01	0.01	0.02	0.00
	L‐W2	0.20	0.00	0.04	0.02	0.01	0.03	0.00
	L‐W3	0.18	0.01	0.04	0.02	0.01	0.03	0.00
	L‐W4	0.13	0.03	0.12	0.02	0.00	0.05	0.00
	L‐W5	0.17	0.00	0.08	0.01	0.00	0.10	0.00
	L‐W6	0.22	0.00	0.30	0.00	0.27	0.06	0.00
	L‐W7	0.18	0.07	0.08	0.03	0.07	0.01	0.00
	L‐W8	0.25	0.01	0.09	0.01	0.06	0.03	0.00
	L‐W9	0.18	0.09	0.07	0.03	0.08	0.02	0.00
	L‐W10	0.11	0.03	0.15	0.02	0.02	0.00	0.00
	L‐W11	0.18	0.02	0.14	0.02	0.02	0.00	0.00
High altitude	H‐W1	0.06	0.06	0.04	0.24	0.00	0.00	0.03
	H‐W2	0.37	0.03	0.12	0.08	0.00	0.00	0.01
	H‐W3	0.02	0.08	0.01	0.41	0.00	0.00	0.06

**Table A9:** Composition of water microbiome at genus level at high and low altitude

Altitude	Samples	Water microbiota composition (relative abundance)					
		Flavobacterium	hgcI clade	Limnohabitans	Sphingomonas	Bosea	Reyranella
Low altitude	L‐W1	0.02	0.11	0.10	0.00	0.00	0.00
	L‐W2	0.04	0.16	0.09	0.00	0.00	0.00
	L‐W3	0.04	0.18	0.06	0.00	0.00	0.00
	L‐W4	0.12	0.16	0.09	0.00	0.00	0.00
	L‐W5	0.08	0.26	0.12	0.00	0.00	0.00
	L‐W6	0.30	0.02	0.04	0.00	0.00	0.00
	L‐W7	0.08	0.00	0.09	0.00	0.00	0.00
	L‐W8	0.09	0.09	0.10	0.00	0.00	0.00
	L‐W9	0.07	0.03	0.07	0.00	0.00	0.00
	L‐W10	0.15	0.00	0.09	0.00	0.00	0.00
	L‐W11	0.14	0.00	0.16	0.00	0.00	0.00
High altitude	H‐W1	0.04	0.00	0.00	0.23	0.03	0.04
	H‐W2	0.12	0.00	0.00	0.06	0.01	0.01
	H‐W3	0.01	0.00	0.00	0.41	0.06	0.06

**Table A10:** Composition of soil microbiome at phylum level at high and low altitude

Altitude	Samples	Soil microbiota composition (relative abundance)						
		Acidobacteria	Actinobacteria	Bacteroidetes	Chloroflexi	Proteobacteria	Planctomycetes	Firmicutes
Low altitude	L‐S1	0.09	0.15	0.02	0.16	0.48	0.03	0.02
	L‐S2	0.09	0.11	0.08	0.14	0.48	0.02	0.03
	L‐S3	0.07	0.26	0.03	0.09	0.44	0.04	0.00
	L‐S4	0.21	0.04	0.01	0.05	0.35	0.07	0.03
	L‐S5	0.18	0.05	0.02	0.05	0.38	0.07	0.08
	L‐S6	0.19	0.05	0.01	0.08	0.46	0.04	0.00
	L‐S7	0.19	0.05	0.01	0.05	0.43	0.05	0.02
	L‐S8	0.15	0.13	0.02	0.06	0.37	0.06	0.05
	L‐S9	0.18	0.09	0.01	0.07	0.36	0.07	0.07
	L‐S10	0.15	0.09	0.02	0.06	0.24	0.08	0.22
	L‐S11	0.21	0.06	0.01	0.05	0.32	0.07	0.14
	L‐S12	0.15	0.05	0.02	0.05	0.28	0.06	0.30
	L‐S13	0.11	0.02	0.04	0.10	0.34	0.03	0.12
	L‐S14	0.11	0.01	0.07	0.08	0.48	0.02	0.03
	L‐S15	0.02	0.03	0.03	0.07	0.51	0.01	0.18
	L‐S16	0.08	0.05	0.03	0.14	0.18	0.01	0.47
	L‐S17	0.05	0.09	0.01	0.04	0.15	0.02	0.50
	L‐S18	0.12	0.03	0.02	0.14	0.21	0.02	0.28
	L‐S19	0.11	0.18	0.04	0.04	0.55	0.02	0.01
	L‐S20	0.06	0.36	0.05	0.05	0.35	0.02	0.00
	L‐S21	0.04	0.05	0.07	0.03	0.48	0.06	0.11
High altitude	H‐S1	0.16	0.15	0.04	0.07	0.39	0.06	0.00
	H‐S2	0.08	0.30	0.08	0.05	0.35	0.04	0.00
	H‐S3	0.13	0.03	0.10	0.06	0.56	0.04	0.00

**Table A11:** Table of composition of soil microbiome at family level at high and low altitude

Altitude	Samples	Soil microbiota composition (relative abundance)					
		Burkholderiaceae	Gemmatimonadaceae	Nitrosomonadaceae	Subgroup 6_norank	Bacillaceae	Chitinophagaceae
Low altitude	L‐S1	0.05	0.01	0.04	0.05	0.00	0.00
	L‐S2	0.08	0.01	0.02	0.06	0.00	0.00
	L‐S3	0.08	0.03	0.13	0.05	0.00	0.00
	L‐S4	0.01	0.03	0.07	0.08	0.02	0.00
	L‐S5	0.02	0.02	0.06	0.07	0.06	0.00
	L‐S6	0.01	0.03	0.09	0.07	0.00	0.00
	L‐S7	0.03	0.02	0.09	0.09	0.01	0.00
	L‐S8	0.03	0.02	0.03	0.09	0.04	0.00
	L‐S9	0.01	0.02	0.05	0.08	0.05	0.00
	L‐S10	0.01	0.02	0.05	0.09	0.10	0.00
	L‐S11	0.02	0.03	0.06	0.12	0.07	0.00
	L‐S12	0.02	0.02	0.04	0.09	0.14	0.00
	L‐S13	0.01	0.03	0.04	0.05	0.06	0.00
	L‐S14	0.04	0.02	0.03	0.04	0.01	0.00
	L‐S15	0.01	0.01	0.06	0.01	0.08	0.00
	L‐S16	0.01	0.02	0.01	0.02	0.21	0.00
	L‐S17	0.03	0.10	0.03	0.02	0.21	0.00
	L‐S18	0.01	0.08	0.02	0.02	0.12	0.00
	L‐S19	0.21	0.03	0.05	0.08	0.00	0.00
	L‐S20	0.14	0.03	0.03	0.04	0.00	0.00
	L‐S21	0.05	0.02	0.04	0.01	0.00	0.00
High altitude	H‐S1	0.06	0.04	0.05	0.07	0.00	0.02
	H‐S2	0.08	0.05	0.04	0.04	0.00	0.04
	H‐S3	0.09	0.01	0.07	0.06	0.00	0.03

**Table A12:** Table of composition of soil microbiome at genus level at high and low altitude

Altitude	Samples	Soil microbiota composition (relative abundance)					
		Subgroup 6_norank	Bacillus	Lactococcus	TRA3‐20_norank	Ellin6067	IMCC26256_norank
Low altitude	L‐S1	0.05	0.00	0.00	0.00	0.00	0.00
	L‐S2	0.06	0.00	0.00	0.00	0.00	0.00
	L‐S3	0.05	0.00	0.00	0.00	0.00	0.00
	L‐S4	0.08	0.02	0.00	0.04	0.00	0.00
	L‐S5	0.07	0.03	0.00	0.04	0.00	0.00
	L‐S6	0.07	0.00	0.00	0.03	0.00	0.00
	L‐S7	0.09	0.01	0.00	0.06	0.00	0.00
	L‐S8	0.09	0.02	0.00	0.02	0.00	0.00
	L‐S9	0.08	0.04	0.00	0.05	0.00	0.00
	L‐S10	0.09	0.09	0.08	0.02	0.00	0.00
	L‐S11	0.12	0.06	0.05	0.05	0.00	0.00
	L‐S12	0.09	0.13	0.12	0.02	0.00	0.00
	L‐S13	0.05	0.06	0.03	0.01	0.00	0.00
	L‐S14	0.04	0.01	0.01	0.01	0.00	0.00
	L‐S15	0.01	0.08	0.08	0.01	0.00	0.00
	L‐S16	0.02	0.19	0.20	0.00	0.00	0.00
	L‐S17	0.02	0.21	0.22	0.00	0.00	0.00
	L‐S18	0.02	0.11	0.13	0.00	0.00	0.00
	L‐S19	0.08	0.00	0.00	0.00	0.00	0.00
	L‐S20	0.04	0.00	0.00	0.00	0.00	0.00
	L‐S21	0.01	0.00	0.00	0.00	0.00	0.00
High altitude	H‐S1	0.07	0.00	0.00	0.00	0.02	0.03
	H‐S2	0.04	0.00	0.00	0.00	0.04	0.02
	H‐S3	0.06	0.00	0.00	0.00	0.03	0.00

**Table A13:** Microbiome with significant differences (Kruskal–Wallis H) in skin and intestinal between high and low elevations (phylum level)

Altitude	Sample	Phylum			
Low altitude	skin	Bacteroidetes***	Firmicutes***	Proteobacteria***	
	gut				
High altitude	skin	Bacteroidetes (NS)	Firmicutes***	Proteobacteria***	Actinobacteria (NS)
	gut				

Note

*p* > .05 marked as “NS”; *p* < .001 marked as “***.”

**Table A14:** Microbiome with significant differences (Mann–Whitney U test) in skin and intestinal of *Bufo gargarizans* between high and low elevations (phylum level)

Altitude	Sample	Phylum			
L‐Bg	Skin	Bacteroidetes (NS)	Firmicutes (NS)	Proteobacteria (NS)	
	Gut				
H‐Bg	Skin	Bacteroidetes (NS)	Firmicutes*	Proteobacteria*	
	Gut				

Note

*p* > .05 marked as “NS”; *p* < .05 marked as “*.”

**Table A15:** Microbiome with significant differences (Kruskal–Wallis) in skin and intestinal between high and low elevations (genus level)

Germ	Sample			
	L‐Skin	L‐Gut	H‐skin	H‐Gut
*Bacteroides*	***		***	
*Parabacteroides*	***		NS	
*Acinetobacter*	***		***	
*Sphingomonas*	***		***	
*Afipia*	***		***	
*Reyranella*	***		NS	
*Mesorhizobium*	***		***	
*Rikenella*	***		***	

Note

*p* > .05 marked as “NS”; *p* < .001 marked as “***.”

**Table A16:** Microbiome with significant differences (Mann–Whitney U test) in skin and intestinal of *Bufo gargarizans* between high and low elevations (genus level)

Germ	Sample			
	L‐S‐Bg	L‐G‐Bg	H‐S‐Bg	H‐G‐Bg
*Bacteroides*	NS		NS	
*Acinetobacter*	*		NS	
*Sphingomonas*	***		NS	
*Caulobacteraceae_Unclassified*	***		NS	
*Odoribacter*	NS		NS	
*Ruminococcaceae_uncultured*	NS		NS	

Note

*p* > .05 marked as “NS”; 0.01 < *p* < .05 marked as “*”, *p* < .001 marked as “***.”
